# Resistance to the herbicide metribuzin conferred to *Arabidopsis thaliana* by targeted base editing of the chloroplast genome

**DOI:** 10.1111/pbi.14490

**Published:** 2024-10-20

**Authors:** Issei Nakazato, Wataru Yamori, Hiroyoshi Matsumura, Yuchen Qu, Miki Okuno, Nobuhiro Tsutsumi, Shin‐ichi Arimura

**Affiliations:** ^1^ Laboratory of Plant Molecular Genetics, Graduate School of Agricultural and Life Sciences The University of Tokyo Tokyo Japan; ^2^ Research Fellow of Japan Society for the Promotion of Science Tokyo Japan; ^3^ Institute for Sustainable Agro‐Ecosystem Services, Graduate School of Agricultural and Life Sciences The University of Tokyo Tokyo Japan; ^4^ Department of Biotechnology College of Life Sciences, Ritsumeikan University Shiga Japan; ^5^ Division of Microbiology, Department of Infectious Medicine Kurume University School of Medicine Fukuoka Japan

**Keywords:** chloroplast genome, base editing, ptpTALECD, metribuzin resistance, *Arabidopsis thaliana*

## Abstract

The chloroplast genome has considerable potential to enhance crop productivity, but it remains underutilized in breeding because it is difficult to modify. This study elucidates the potential of recently developed chloroplast‐targeted C‐to‐T base editors in facilitating the use of the chloroplast genome for crop breeding. The herbicide metribuzin interferes with photosynthesis by binding to the D1 protein of photosystem II, encoded by the chloroplast genome. Naturally occurring D1 mutants with V219I or A251V substitutions are known to have resistance to some herbicides including metribuzin. Here, using the base editors, we introduced these substitutions and showed that the A251V single mutation and the V219 & A251V double mutations conferred significant metribuzin resistance to *Arabidopsis thaliana*. The V219I & A251V double mutants exhibited increased metribuzin resistance and grew better than the A251V single mutants. Furthermore, the double mutants grew as well as wild‐type plants in the absence of metribuzin. The single and double mutants, which are a challenge to obtain through traditional mutagenesis and crossbreeding methods, can be relatively easily generated using C‐to‐T base editors. In view of the conservation of V219 and A251 across numerous species, C‐to‐T base editing can potentially confer metribuzin resistance to a wide range of crops. Compared to nuclear genes, chloroplast genes are also less likely to spread into wild populations. Our findings suggest that chloroplast‐targeting C‐to‐T base editors will find many roles in future crop breeding efforts.

## Introduction

Modification of photosynthesis‐related genes encoded in the chloroplast (plastid) genome has the potential to improve crop yield. However, efforts to modify the chloroplast genome in crop breeding by conventional crossing methods have been limited. This can be partly attributed to the uniparental inheritance of the chloroplast genome in many species (Zhang *et al*., 2003), which precludes recombination and thus impedes the generation of novel combinations of parental alleles. Incorporating foreign genes into the chloroplast genome, known as chloroplast transformation (Svab *et al*., 1990), is applicable to only a limited number of species (Rascón‐Cruz *et al*., 2021). Moreover, transformants are classified as genetically modified organisms (GMOs), whose use is subject to restrictions in some countries. Recently reported methods for targeted single‐base editing in the chloroplast genome (Kang *et al*., 2021; Li *et al*., 2021; Mok *et al*., 2022; Nakazato *et al*., 2021, 2023; Wang *et al*., 2024; Zhang *et al*., 2024; Zhang and Boch, 2023; Zhou *et al*., 2024), which avoid the limitations associated with chloroplast transformation, have the potential to unlock the chloroplast genome for future crop breeding, but so far these base editing methods have not been used to improve agronomic traits.

Herbicides increase crop production by impeding the growth of weeds, but herbicides can also cause crops to wither. So, one approach has been to generate herbicide‐resistant crops through modifications of the nuclear genome (Han and Kim, 2019; Jin *et al*., 2022; Owen and Zelaya, 2005; Pandian *et al*., 2022). However, introducing herbicide resistance genes into the nuclear genome can potentially spread into the environment via pollen, which may lead to the acquisition of herbicide resistance by wild relatives. On the other hand, genetic information in the chloroplast genome is deemed more controllable, primarily due to its maternal inheritance in many species resulting in a lower likelihood of dissemination via pollen (Azhagiri and Maliga, 2007; Chung *et al*., 2023). Another advantage is that when a maternal line harbours a herbicide‐resistant mutation in the chloroplast genome, all progeny will inherit the herbicide resistance, whereas if it carries a heterozygous or incompletely dominant mutation in the nuclear genome, only some of the progeny will inherit the trait.

Herbicides classified under groups 5 and 6 (https://hracglobal.com/) interfere with photosynthesis by binding to the D1 protein of photosystem II (PSII), which is encoded by the chloroplast gene *psbA*. Some amino acid polymorphisms in D1 predictably interfere with this binding, and thus confer resistance to the herbicides (Davis *et al*., 2020; Lu *et al*., 2019; Oettmeier, 1999). Two of these polymorphisms, V219I and A251V that confer resistance to herbicides belonging to these groups across multiple organisms (Davis *et al*., 2020; Dupraz *et al*., 2016; Mengistu *et al*., 2000, 2005; Oettmeier, 1999; Schwenger‐Erger *et al*., 1993; Thiel and Varrelmann, 2014), can be introduced by C‐to‐T base editing. Specifically, these two polymorphisms can confer resistance to metribuzin (Oettmeier, 1999). This study focuses on modifying the chloroplast genome through C‐to‐T base editing to enhance an agronomic trait. Initially, we introduced the V219I and A251V mutations into the D1 protein of *Arabidopsis thaliana* ecotype Columbia‐0 (Col‐0), an ecotype that is known to be resistant to chloroplast transformation (Yu *et al*., 2017). The introduction of these mutations was accomplished using efficient C‐to‐T base editors, ptpTALECD and ptpTALECD_v2 (Nakazato *et al*., 2021, 2023). Subsequently, we evaluated the mutants' resistance to metribuzin.

## Results

### V219 and A251 are conserved and their mutations for resistance to metribuzin

Among 275 species that we investigated, from photosynthetic bacteria to angiosperms, all except herbicide‐resistant weeds exhibited V219 and A251 residues in the D1 protein (Table S1). The codons corresponding to V219 and A251 are conserved among *A. thaliana* and 12 other crops that we investigated (Figure S1). These species are, thus, candidates for V219I and A251V substitutions. Furthermore, *A. thaliana* was reported to be sensitive to metribuzin (Ratsch *et al*., 1986). Consequently, we selected *A. thaliana* for the test.

### Modification of *psbA* by chloroplast‐targeted base editors

**Figure 1:**
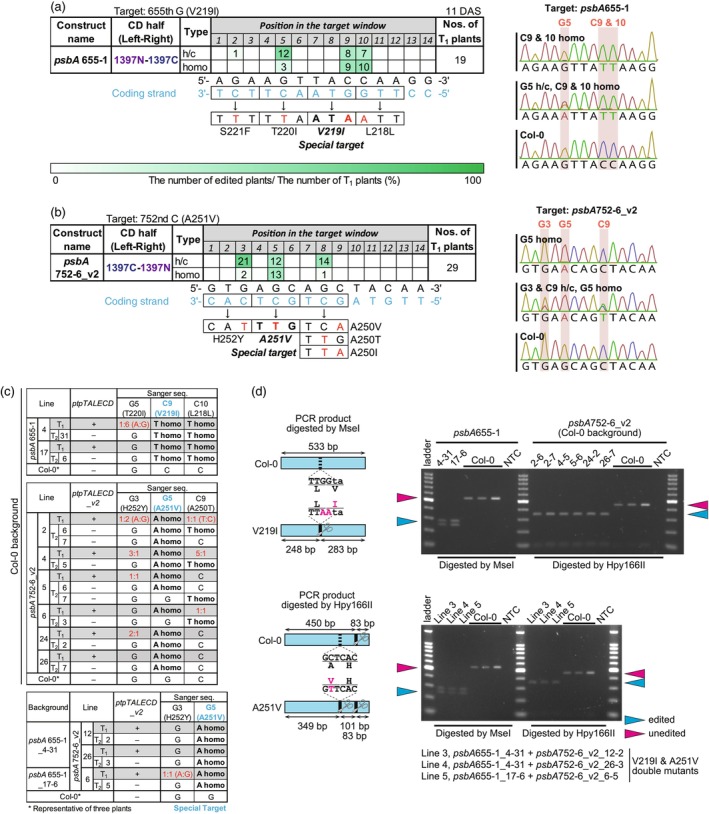
Introduction of V219I and A251V mutations in the D1 protein encoded by *psbA*. (a, b) Targeted base editing in *psbA*. Targets of a and b were 655th G (V219I) and 752nd C (A251V) respectively. Numbers of T_1_ plants that had edited bases at each position in the target windows and predicted amino acid substitutions are shown in tables. Editing efficiencies are shown by the intensity of the green colour. Representative data from Sanger sequencing in the target windows of T_1_ plants are shown on the right side (a, *psbA*655‐1; b, *psbA*752‐6_v2). h/c, heteroplasmically and/or chimerically substituted; homo, homoplasmically substituted. The data for the plants with ptpTALECD targeting *psbA*655‐1 were derived from Nakazato *et al*., 2023. Data for plants with each of the all constructs examined are shown in Figure S2. (c) Inheritance of the edited bases by T_2_ generation. CTP‐TALn is a part of the open reading frame of the base editor gene. (d) CAPS analysis of T_2_ plants whose progenies are subjected to metribuzin resistance test. NTC, non‐template control. ladder, 100 bp DNA Ladder (Takara). [Correction added on 6 November 2024, after first online publication: the upper scale bar in the Figures 1, 2, 3, 4 are removed in this version.]

To accomplish the V219I and A251V substitutions, we constructed binary vectors, each of which encodes a pair of chloroplast‐targeted C‐to‐T base editors [ptpTALECD or ptpTALECD_v2 (Nakazato *et al*., 2021, 2023), Figure S2a]. Subsequently, these vectors were individually integrated into the nuclear genome of *A. thaliana* by floral dipping (Clough and Bent, 1998). The genotypes of the transformants were determined by Sanger sequencing, and the genotypes of some T_2_ plants were further investigated by next‐generation sequencing (NGS). Both vectors for the V219I mutation appeared to homoplasmically introduce (i.e. to introduce to all copies of the chloroplast genome within a cell) DNA mutations that cause the V219I mutation in the T_1_ generation (Figure S2b). The number of chloroplast genomes in a cell can range from hundreds to thousands (Zoschke *et al*., 2007). Figure 1a shows the result of base editing by one vector (*psbA*655‐1). This vector did not homoplasmically edit non‐target bases compared to the other vector (Table S2). Similarly, one of the 12 vectors for the A251V mutation appeared to homoplasmically introduce a DNA mutation that causes the A251V mutation in the T_1_ generation (Figure S2c). The A251V mutation seemed to be homoplasmically introduced in 13 of 29 T_1_ plants by this vector (*psbA*752‐6_v2, Figure 1b). The allele patterns of all the T_1_ plants in which the V219I and A251V mutations were attempted (Tables S2 and S3 respectively) confirmed that the only homoplasmic mutations in some plants were the target mutation and synonymous mutations. We investigated whether introduced mutations were inherited by the next generation. The V219I mutation was inherited by T_2_ progenies across all three examined lines (Table S4, left panel), while the A251V mutation was inherited by T_2_ progenies in six of the examined eight lines (Table S4, right panel). The nuclei of some of the base‐edited T_2_ progenies lacked the base editor gene (Table S4), suggesting that no further mutations would occur in the chloroplast genome in these plants and their progenies. So only already introduced mutations would affect phenotypes of these plants. T_3_ progenies of 10 of these plants (Table S5) were tested for metribuzin resistance and subjected to other phenotyping assays. The genotypes of the parental T_2_ plants were determined by Sanger sequencing (the results are summarized in Figure 1c) and by Cleaved Amplified Polymorphic Sequences (CAPS) assays (Figure 1d). Figure 1d shows the locations of cleavage (left) and the products of the cleavage (right). Together, these figures suggest that the T_2_ plants had either a V219I homoplasmic mutation or an A251V homoplasmic mutation. We also generated V219I & A251V double mutants by using T_3_ plants of the V219I single mutants as recipients. The data in Table S6 suggest that the A251V mutation was homoplasmically introduced in the V219I mutants. Two lines of T_2_ plants, whose siblings stably inherited the double mutations (Table S4, highlighted in green), and three lines of T_3_ plants were also tested for metribuzin resistance. We confirmed that the parental T_2_ plants of these three lines had V219I & A251V double mutations by Sanger sequencing (bottom table of Figure 1c) and CAPS assay (bottom‐right panel of Figure 1d). To make the following text and figures more readable, we assigned some of the lines shortened names (Table S5).

### A251V confers metribuzin resistance

**Figure 2:**
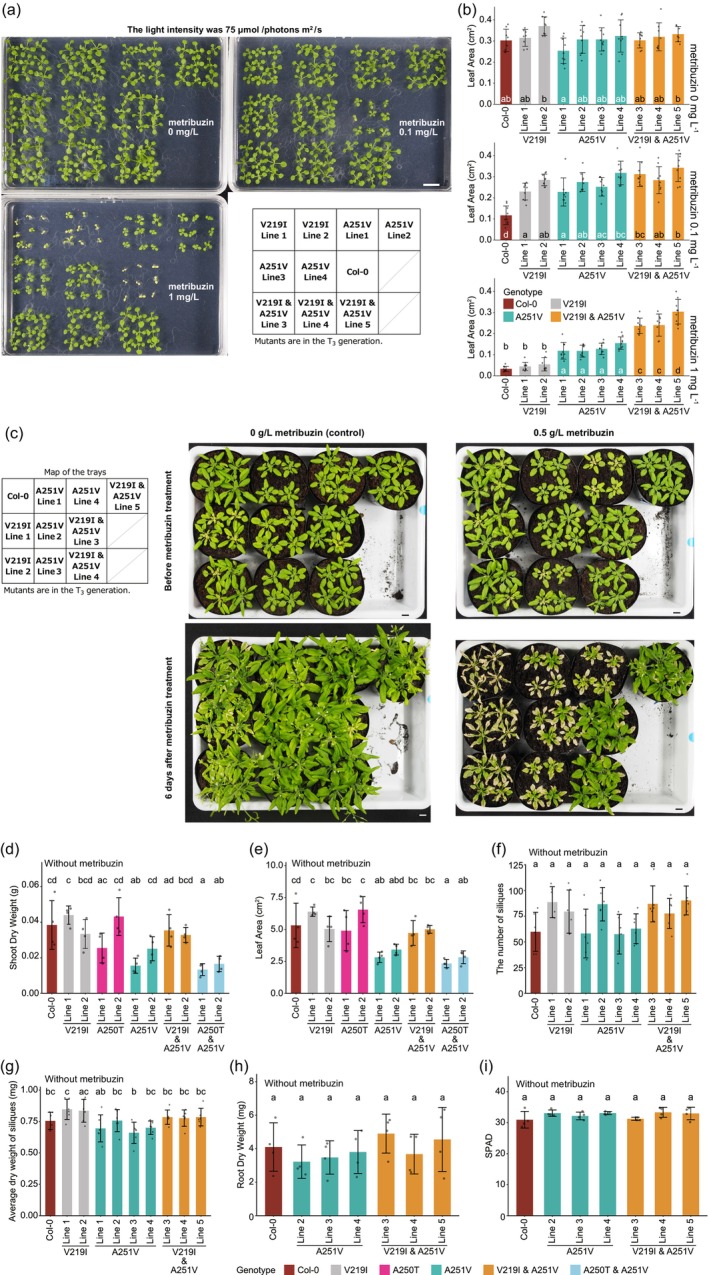
Growth of *psbA* mutants in the presence or absence of metribuzin. (a) Appearance of *psbA* mutants at 12 days after stratification (DAS) cultivated on 1/2 MS plates containing 0, 0.1 or 1 mg/L of metribuzin under the light intensity of 75 μmol/photons m^2^/s. Nine seeds were sown on one plate for each line. Bar = 1 cm. (b) Leaf areas of the plants shown in (a). In Figure b, d–i, different letters indicate significantly different values (*P* < 0.05, Tukey–Kramer multiple comparison test), and error bars represent standard deviation. *n* (the number of plants) = 8 or 9. (c) Metribuzin treatment on *psbA* mutants in soil. Metribuzin was sprayed on the plants at 25 DAS. Photos at 25 DAS (before metribuzin treatment) and 6 days after metribuzin treatment are shown. Bars = 1 cm. (d–i) Shoot dry weight (d), leaf area (e), the number of siliques (f), the average dry weight of siliques (g), root dry weight (h) and the chlorophyll content (i, SPAD values) of the plants cultivated in soil. All the T_2_ plants (V219I & A251V Lines 1 and 2) were confirmed to have the V219I & A251V double mutations and not to have *ptpTALECD_v2* in the nuclear genome by PCR analyses. *n* (the number of plants) = 4 (d, e, h, i) and 6 (f, g).

To assess the resistance of the generated *psbA* mutants to metribuzin, we cultivated them on 1/2 MS agar plates with 0, 0.1 or 1 mg/L metribuzin (Figure 2a, Figure S3a). We selected these concentrations because they inhibited the growth of wild‐type plants in a gradient manner in the preliminary experiment. The plates were divided into 12 regions, each containing a mutant or a wild‐type plant as shown in Figure 2a and Figure S3b. Under the intensity of 75 μmol/photons m^2^/s and in the absence of metribuzin, all the mutants displayed green leaves similar to those of wild‐type plants in duplicated experiments (Figure S3a, left plate; Figure 2a, top‐left plate). These results suggest that the introduced mutations did not greatly impede growth under these conditions.

In the presence of 1 mg/L metribuzin, wild‐type plants and the V219I and A250T mutants exhibited poor growth, while the A251V single mutants and the A250T & A251V and V219I & A251V double mutants were not noticeably affected in duplicated experiments (Figure S3a, right plate; Figure 2a, bottom‐left plate). In addition, in the presence of 1 mg/L metribuzin, the leaf areas of the resistant lines (green and orange bars) were significantly larger than those of wild‐type plants (brown bar). Furthermore, NGS analyses of the seven T_2_ plants (A251V mutants and V219I & A251V double mutants) whose progenies displayed metribuzin resistance in the assay revealed that the target mutations were present in 99.7% to 100.0% of NGS reads in all the plants (Figure S4a). Only one individual (A251V Line 1) had an off‐target mutation in the chloroplast genome (Figure S4a). These analyses also identified a bystander error (i.e. a mutation around the target) in three of the seven plants (V219I & A251V Lines 3–5; Figure S4a), but this mutation was synonymous (L218L) so was predicted not to affect the strength of metribuzin resistance. A251V Line 1 had an off‐target mutation in the single intron of *petD* (Figure S4a, bottom line). A quantitative reverse transcription PCR (qRT‐PCR) analysis indicated that this mutation resulted in a decrease in mature *petD* mRNAs (Figure S4b). These results indicate that the A251V mutation confers metribuzin resistance to *A. thaliana*.

In the presence of 0.1 mg/L of metribuzin, V219I mutants grew better (Figure 2a) and had larger leaf areas (Figure 2b) than did wild‐type plants, indicating that V219I mutants were more resistant to metribuzin than the wild‐type plants. Figure 2b shows the growth of the plants. In the absence of metribuzin, the leaf areas of the V219I & A251V double mutants were similar to those of the A251V mutants (Figure 2b, top panel), while in the presence of 1 mg/L of metribuzin, they were significantly larger than those of the single mutants (Figure 2b, bottom panel). These results indicate that V219I & A251V double mutants are more resistant to metribuzin than the A251V single mutants.

Next, we investigated the effects of spraying 0.5 g/L metribuzin at a rate of 100 mL/m^2^ on *psbA* mutants. This concentration and rate are comparable to recommended dosages for cultivating metribuzin‐applicable crops (https://cropscience.bayer.jp/ja/home/product/detail/product14683.php). This concentration is 500 times higher than that employed for the metribuzin resistance test on MS plates (1 mg/L). On the MS plates, plants continuously absorbed metribuzin, likely making the lower concentration sufficient for selecting resistant lines. Indeed, culturing tomato seedlings in a liquid medium containing 0.25 ppm (approximately 0.25 mg/L) of metribuzin was sufficient for selecting metribuzin‐resistant lines (Souzamachado *et al*., 1982). We note that Brassicaceae species, including *A. thaliana*, are not among the crops recommended for metribuzin treatment (https://www.katyayaniorganics.com/product/metribuzin‐70‐wp‐metzin/). Before the spray treatment, all individuals exhibited green leaves (Figure 2c, top panels). Six days after the treatment, all plants in the control group (plants sprayed with water) exhibited green leaves (bottom‐left panel in Figure 2c). On the other hand, when the plants were sprayed with metribuzin, the wild‐type plants and V219I mutants withered and the A251V mutants partially withered, while the V219I & A251V double mutants were not noticeably affected (Figure 2c, bottom‐right panel). Similar results were obtained in six replicated experiments (Figure S5). The observed differences in metribuzin resistance between lines of the same genotype may be attributed to variations in the amount of metribuzin adhering to individual plants. In this experiment, metribuzin was applied via spraying, which likely led to an uneven distribution of the herbicide due to the complex shape of the above‐ground part of plants. Nevertheless, the results consistently demonstrated through experimental replicates that Col‐0 perished following metribuzin spraying, whereas the V219I & A251V double mutants survived. In the control group of replicate 3, an individual of V219I Line 2 and two individuals of A251V Line 3 had chlorotic or withered leaves (Figure S5b, top‐left panel), which were present before spraying and therefore not attributable to the treatment. These results suggest that V219I & A251V double mutations conferred strong metribuzin resistance.

### Growth and chlorophyll fluorescence tests of *psbA* mutants

In soil conditions, A251V mutants and A250T & A251V double mutants had slightly or significantly smaller shoot dry weights (Figure 2d, Figure S6a) and leaf areas (Figure 2e, Figure S6b–e) than did wild‐type plants. However, shoot dry weights of the V219I & A251V double mutants were comparable to those of the wild‐type plants (Figure 2d). Leaf areas of the V219I & A251V double mutants were comparable to or slightly smaller than those of wild‐type plants, yet larger than those of A251V mutants and A250T & A251V double mutants (Figure 2e, Figure S6b–e). The number of siliques (Figure 2f), the average dry weight of siliques (Figure 2g), root dry weights (Figure 2h) and chlorophyll content (SPAD values, Figure 2i) did not significantly differ among wild‐type plants, A251V mutants and V219I & A251V double mutants. When cultivated on sucrose‐containing 1/2 MS agar plates under the light intensity of 75 μmol/photons m^2^/s, the leaf areas of A251V mutant lines and V219I & A251V double mutant lines were similar to those of wild‐type plants (Figure 2b, the top panel). These results suggest that, in soil conditions, the A251V mutation had an inhibitory effect on growth. In addition, the V219I single mutation did not enhance growth but its combination with the A251V mutation partially mitigated the inhibitory effect of the A251V mutation.

**Figure 3:**
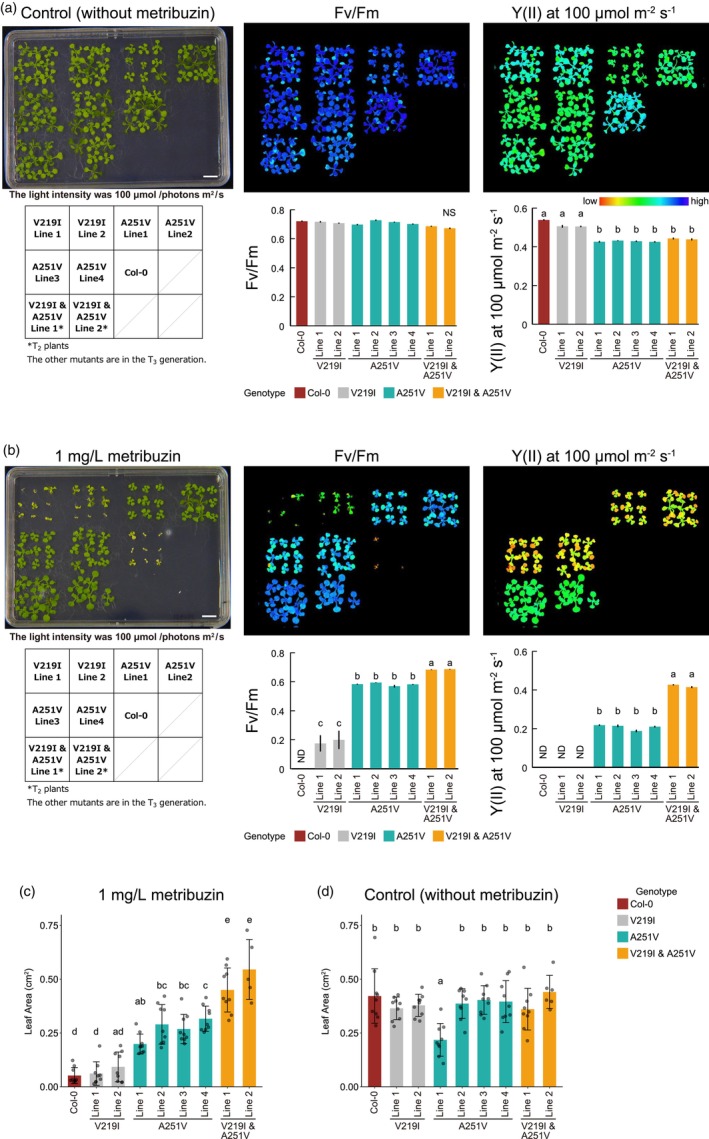
Photoinhibition and photosynthetic electron transport in *psbA* mutants in the absence or presence of metribuzin. (a, b) The degree of photoinhibition and the photosynthetic electron transport rate at 100 μmol/photons m^2^/s were analysed at 12 days after stratification in the absence (a) or presence (b) of metribuzin. Data are means ± standard errors. *n* (the number of plants) = 5–9. Different letters indicate significantly different values (*P* < 0.05, Tukey–Kramer multiple comparison test). ND, not detected; NS, not significant. See also Figure S7. (c, d) Leaf areas of plants shown in (a) and (b). Different letters indicate significantly different values (*P* < 0.05, Tukey–Kramer multiple comparison test). *n* (the number of plants) = 5–9. All the T_2_ plants (V219I & A251V Lines 1 and 2) were confirmed to have the V219I & A251V double mutations and most of the T_2_ plants (except for one plant of V219I & A251V Line 1 on the metribuzin‐containing medium) were confirmed not to have *ptpTALECD_v2* in the nuclear genome by PCR analyses.

We next evaluated the photosynthetic activity of the mutants with chlorophyll fluorescence analysis of the *psbA* mutants (V219I mutants, A251V mutants, and V219I & A251V double mutants) cultivated on sucrose‐containing 1/2 MS plates with or without metribuzin (Figure 3a,b). As observed under the light intensity of 75 μmol/photons m^2^/s, V219I & A251V double mutants had significantly larger leaf areas than wild‐type plants and A251V mutants in the presence of 1 mg/L metribuzin under the light intensity of 100 μmol/photons m^2^/s (Figure 3c). Similarly, in the absence of metribuzin, the leaf areas of the double mutant were comparable to those of wild‐type plants (Figure 3d). In the absence of metribuzin, no discernible differences in Fv/Fm were observed between wild‐type plants and the mutants (Figure 3a). However, under lower light intensity (100 μmol/photons m^2^/s), plants harbouring the A251V mutation exhibited reduced quantum yields of PSII [Y(II)], which is indicative of lowered photochemical efficiency of electron transfer through PSII (Figure 3a; Figure S7a, lower left panel), in agreement with a previous study (Rochaix and Erickson, 1988). On the other hand, under higher light intensity (300 μmol/photons m^2^/s), no discernible differences in Y(II) were observed between wild‐type plants and the mutants (Figure S7a, lower left panel). In the presence of metribuzin, wild‐type plants exhibited withering and a lack of chlorophyll fluorescence from leaves (Figure 3b). The V219I mutants displayed chlorosis and substantial reductions in Fv/Fm (Figure 3b). In contrast, the A251V mutants could survive and showed high Fv/Fm values, while the V219I & A251V double mutants showed higher Fv/Fm values than did the A251V mutants (Figure 3b). Moreover, in the presence of metribuzin, across all light intensities, compared to A251V mutants, the V219I & A251V double mutants showed higher Y(II) (Figure S7b, bottom‐left panel), lower Y(NPQ) (a parameter indicative of thermal dissipation of excitation energy absorbed for photosynthesis; Figure S7b, bottom‐middle panel) and lower Y(NO) (a parameter indicative of photoinhibition at PSII; Figure S7b, bottom‐right panel). When cultivated on soil, Fv/Fm of A251V mutants and V219I & A251V double mutants were comparable to those of wild‐type plants (Figure S8a). In soil conditions, these mutants showed slightly or significantly lower Y(II) (Figure S8b) and electron transfer rate (Figure S8c) than wild‐type plants. These results suggest that the A251V mutation and the V219I & A251V double mutations decreased the electron transfer rate of PSII in the absence of metribuzin. Additionally, these mutations, particularly the V219I & A251V double mutations, appeared more effective at averting a reduction in the electron transfer rate of PSII induced by metribuzin.

### A251V is predicted to inhibit metribuzin from binding to D1

**Figure 4:**
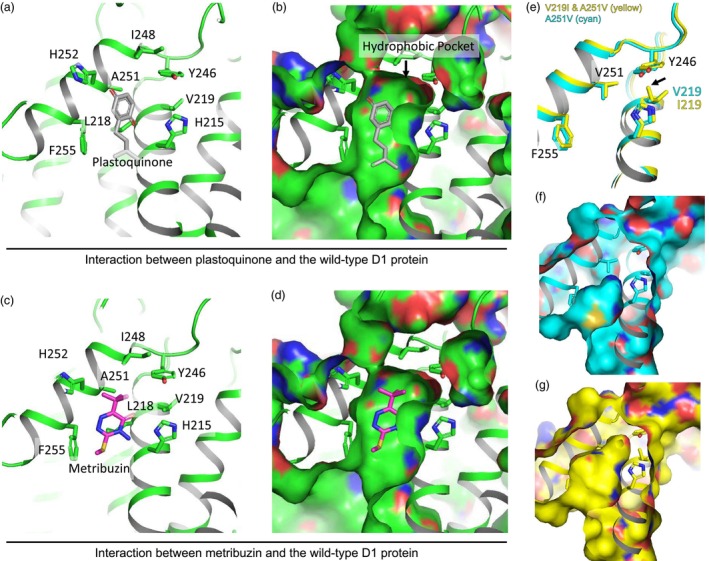
Interaction between metribuzin or plastoquinone and the D1 protein. (a) Hydrophobic residues around the benzoquinone group of plastoquinone in the Arabidopsis PSII resolved by cryo‐electron microscopy (Graça *et al*., 2021). (b) Molecular surface of the residues shown in (a). The benzoquinone group is surrounded by the hydrophobic pocket. (c) Binding model between metribuzin and the hydrophobic pocket. (d) Molecular surface of the hydrophobic pocket surrounding metribuzin. (e) The superimposed image of the hydrophobic pocket of V219I & A251 double mutants (yellow) and A251V mutants (cyan). I219 in the double mutants lifts Y246 (indicated by an arrow). (f, g) Molecular surface of the hydrophobic pocket of A251V mutants (cyan, f) and V219I & A251V double mutants (yellow, g). The structures of the D1 protein were predicted by using AlphaFold2.

To better understand the effect of the mutants on metribuzin resistance, we constructed models of metribuzin binding to the wild‐type D1 protein via docking simulations, using AutoDock Vina (Trott and Olson, 2010). The structure of the wild‐type D1 protein was referenced from a previous study (Graça *et al*., 2021) (PDB id: 7OUI). Metribuzin is suggested to compete with native plastoquinone at the Q_B_ site of D1 (Battaglino *et al*., 2021; Lu *et al*., 2019). Based on the structure of Arabidopsis PSII obtained by cryo‐electron microscopy (Graça *et al*., 2021) (PDB id: 7OUI), the benzoquinone group of the plastoquinone resides within a hydrophobic pocket formed by the side chains of F255, H252, A251, I248, L218 and V219 of the D1 protein (Figure 4a,b). In our docking model, the tertiary‐methyl group of metribuzin binds to the hydrophobic pocket (Figure 4c,d), as in a previous docking model of metribuzin to the wild‐type D1 protein of pea (Battaglino *et al*., 2021). Metribuzin is close to the side chain of A251. Replacing A251 with a Val is presumed to induce steric clashes between metribuzin and the hydrophobic pocket, thus preventing the binding of metribuzin. Additionally, the A251V substitution is anticipated to introduce steric clashes between plastoquinone and the hydrophobic pocket, thereby impeding electron transport in PSII. We also predicted the structures of the D1 protein of A251V mutants and V219I & A251V double mutants using ColabFold v1.5.3 (AlphaFold2 using MMseqs2) (Jumper *et al*., 2021; Mirdita *et al*., 2022). Confidences of the structure around the hydrophobic pocket based on predicted LDDT scores were confident. Superimposition of the predicted D1 proteins from V219I & A251V double mutants and A251V mutants suggest that the V219I substitution elevates the side chain of Y246 (Figure 4e), which slightly expands the hydrophobic pocket [Figure 4f (A251V), g (V219I & A251V)]. Expansion of the pocket might facilitate the accommodation of plastoquinone while still inhibiting the binding of metribuzin. This structural insight collectively provides a plausible explanation for why the V219I & A251V double mutants grew better than the A251V mutants in soil conditions. Although further structural studies are needed to confirm this model, our model provides support for the positive effect of A251V on metribuzin resistance and the superior growth of the V219I & A251V double mutants in soil compared to A251V mutants.

## Discussion

The A251V mutation and the V219I & A251V double mutations in the D1 protein clearly conferred metribuzin resistance to *A. thaliana* (Figures 2a, 3b). The V219I mutation conferred metribuzin resistance to *Chlamydomonas*, *Poa annua* and *Kochia scoparia* (Mengistu *et al*., 2000, 2005; Oettmeier, 1999), but its impact on *A. thaliana* was comparatively weak (Figure 2b). Our results are consistent with a previous report that the V219I mutation was less effective than the A251V mutation in conferring metribuzin resistance to *Amaranthus* (Davis *et al*., 2020).

In the absence of metribuzin, the A251V mutants and V219I & A251V double mutants showed lower Y(II) than V219I mutants and wild‐type plants (Figure 3a). This is consistent with a previous study of *Chlamydomonas reinhardtii* (Rochaix and Erickson, 1988) in which the A251V mutation decreased the electron transfer rate while the V219I mutation had no effect. The V219I & A251V double mutants, when grown in soil and in the absence of metribuzin, grew better than the A251V single mutants in terms of dry weight (Figure 2d, Figure S6a) and leaf area (Figure 2e, Figure S6b–e). Furthermore, these double mutants showed stronger metribuzin resistance than the A251V mutants (Figure 2c, Figure S5). In view of the conservation of V219 and A251 across photosynthetic organisms (Table S1), the V219I & A251V double mutant appears to be better at conferring metribuzin resistance than the A251V single mutant.

Employing base editors to modify the chloroplast genome has several advantages over obtaining plants from natural environments that have double mutations in the chloroplast genome. While the former is relatively easy and time‐efficient, the latter is difficult due to the slow mutational rate of the chloroplast genome (Wolfe *et al*., 1987). In addition, even if such double mutants are obtained from natural environments, integrating them into an elite cultivar (serving as the paternal line) through continuous backcrosses would be costly and time‐consuming. Indeed, eight continuous backcrosses were required to incorporate a naturally occurring mutation in the chloroplast genome of a *Brassica* weed into rapeseed (Gressel and Ben‐Sinai, 1985). Such backcrosses also risk transferring undesired single nucleotide polymorphisms (SNPs) in the chloroplast genome from the weed. On the other hand, we successfully introduced the desired double mutations (V219I & A251V) without undesired additional SNPs or off‐targets into wild‐type plants within four generations. Another potential problem with such continuous backcrosses is that the chloroplast and mitochondrial genes coming from the maternal line, when combined with the nuclear genes coming from the paternal line, could impede growth. This phenomenon is called nuclear‐cytoplasmic incompatibility or cytonuclear incompatibility (Postel and Touzet, 2020). Chemical mutagens like ethyl methanesulfonate can also induce base substitutions in the chloroplast genome (McMurray *et al*., 2019). However, introducing two desired mutations together without concomitantly introducing undesired mutations is almost impossible. Moreover, the selection of herbicide‐resistant lines from the mutant population requires substantial manpower and extensive field resources (McMurray *et al*., 2019). The base editing method will bypass these challenges. An alternative approach to introduce herbicide resistance mutations into the chloroplast genome is to select mutagenized or fused cultured cells in the presence of herbicides (Csépl'ó *et al*., 1985; Menczel *et al*., 1986). Using base editors, these identified mutations can be precisely introduced into crops, thereby conferring herbicide resistance. The base editors (ptpTALECD and ptpTALECD_v2) and the mutations (V219I and A251V) were previously reported. However, this study will contribute to plant biotechnology for three reasons. First, we demonstrated that these mutations could be introduced without off‐target mutations into a novel species that is recalcitrant to conventional methods. Second, we found a practical advantage wherein V219I & A251V double mutants showed stronger resistance to metribuzin and grew better than A251V mutants, while the double mutants grew as well as wild‐type plants. The double mutations likely conferred metribuzin resistance to cultured cells of *Chenopodium rubrum* (Schwenger‐Erger *et al*., 1993), a species distant from *A. thaliana*, but their impact on growth was not assessed. Our findings, in conjunction with this previous study, suggest that these double mutations are likely to confer metribuzin resistance to multiple species. Third, we also showed that these mutations can be similarly introduced into various crops (Figure S1).

Generating herbicide‐resistant crops by editing the chloroplast genome has two additional advantages. First, expanding the range of crops resistant to commercially available herbicides can reduce the need for developing new herbicides, and thereby avoid the expenses associated with their development and approval. Second, the metribuzin‐resistant plants generated in this study lacked base‐editor genes in the nucleus and were presumed to be null segregants, which are devoid of foreign DNAs in their genomes. Null segregants are categorized as non‐GMOs in some countries, so farmers have to satisfy fewer rules to cultivate them than they do to cultivate GMOs. In addition, as described above, conferring herbicide resistance to crops by modifying the chloroplast genome avoids the possibility of contaminating wild genetic resources. Furthermore, using genome editing methods is less time‐consuming and less costly than conventional breeding techniques. Thus, again, generating herbicide‐resistant crops by modifying the chloroplast genome with a genome editing technology holds substantial significance concerning economic feasibility, crop production and conserving wild genetic resources.

## Conclusion

The chloroplast genome has been underutilized for crop breeding despite its potential advantages. This study contributes to filling this gap by demonstrating the efficacy of modifying the chloroplast genome by base editors for conferring metribuzin resistance to plants. Chloroplast‐targeted base editors can be powerful tools for modifying the chloroplast genome for crop breeding.

## Materials and methods

### Sequence analysis of the D1 protein

*psbA* sequences were obtained from reference genomes (Table S1) and compared using Geneious Prime (version 2023.2.1).

### Vector construction

Binary vectors that encode ptpTALECD and ptpTALECD_v2 were assembled as described previously (Nakazato *et al*., 2021, 2023). TALE domains were assembled with the Platinum Gate TALEN kit [Addgene ID Kit #1000000043, (Sakuma *et al*., 2013)] and the plasmids that we previously made (Addgene ID #171723–171736, 191579–191598, 191603–191606, 191611–191614, and 199797–199804). The vector construction procedure is divided into three steps. In the first step, one to four TALE repeats were assembled into a single plasmid. In the second step, multiple plasmids made in the first step were assembled into a single plasmid that encodes the open reading frame of the left or right hand of ptpTALECD or ptpTALECD_v2. In this step, we used the entry vectors (Addgene ID #171724–171735, 191579–191598, 191603–191606, 191611–191614, and 199797–199804) instead of those in the Platinum Gate TALEN kit. In the third step, two plasmids made in the second step and two other plasmids (Addgene ID #171723 and 171736) that encode promoters for the base editor and selection marker genes were assembled into a single binary vector with the Multisite Gateway system (Thermo Fisher Scientific, Waltham, MA). The sequences that the TALE arrays bind to are listed in Table S7.

### Plant materials for agrobacterium‐mediated transformation and genotyping

Seeds of *Arabidopsis thaliana* ecotype Columbia‐0 (Col‐0) and T_2_ transformants were sown on 1/2 Murashige‐Skoog (MS) medium (pH = 5.9) containing 2.3 g/L Murashige and Skoog Plant Salt Mixture (Fujifilm Wako, Osaka, Japan), 500 mg/L MES (Dojindo, Tokyo, Japan), 10 g/L Sucrose (Fujifilm Wako), 1 mL/L Plant Preservative Mixture (Plant Cell Technology, Washington, DC), 1 mL/L 1000× MS vitamin solution and 7 g/L Agar (Fujifilm Wako). 1000× MS vitamin solution consists of 100 g/L myo‐Inositol (Fujifilm Wako), 2 g/L Glycine (Fujifilm Wako), 500 mg/L Nicotinic acid (Fujifilm Wako), 500 mg/L Pyridoxine hydrochloride (Sigma‐Aldrich) and 100 mg/L Thiamine hydrochloride (Fujifilm Wako). Seeds of T_1_ transformants were sown on 1/2 MS medium containing 125 mg/L Claforan. Seedlings were transferred to Jiffy‐7 (Jiffy Products International, Zwijndrecht, the Netherlands) at 3 weeks' age. Plants were grown at 22 °C under the long‐day condition (16‐h light and 8‐h dark). Each binary vector that encodes ptpTALECD or ptpTALECD_v2 was introduced to the nuclear genome of Col‐0 and V219I mutants by floral dipping (Clough and Bent, 1998).

### Genotyping of transformants

T_1_ transformants were selected by GFP fluorescence as described previously (Nakazato *et al*., 2023). Briefly, seeds that exhibited GFP fluorescence expressed by the binary vector (Nakazato *et al*., 2021, 2022, 2023; Shimada *et al*., 2010) were selected and the presence of T‐DNA was checked by PCR. The selected T_1_ plants were used for genotyping the chloroplast genome by Sanger sequencing. PCRs for Sanger sequencing (Eurofins Genomics) were performed using KOD one PCR Master Mix (TOYOBO) with DNA that was roughly extracted by co‐incubating a leaf or a cotyledon with TE Buffer [100 mm Tris–HCl (pH 9.5) and 10 mm EDTA (pH 8.0) in diluted water] at 98 °C for 15 min. PCR products were purified using FastGene Gel/PCR Extraction Kit (Nippon Genetics, Tokyo, Japan). Primers used for PCRs are listed in Table S8.

T_2_ seeds that did not show GFP fluorescence (i.e. seeds that were expected to lack the base editor gene in the nuclear genome) were selected and sown. The absence of the *ptpTALECD_v2* gene was confirmed by PCR using the primers shown in Table S8. T_2_ plants were genotyped in the same way that T_1_ plants were genotyped. Genotypes of the T_2_ plants whose progenies were tested for herbicide resistance were also investigated by the CAPS assay. For this, 1 μL of PCR products was co‐incubated with 7.5 μL of distilled water, 1 μL of rCutSmart Buffer (New England Biolabs, Ipswich, MA) and 0.5 μL of MseI or Hpy166II (New England Biolabs) at 37 °C for 72 h. The digested PCR products were electrophoresed on 2% agarose gels under 100 kV for 30 min.

### Metribuzin resistance test on 1/2 MS medium

Seeds were sown on 1/2 MS medium without plant preservative mixture (PPM, see ‘Plant materials for Agrobacterium‐mediated transformation and genotyping’) containing 0, 0.1 or 1 mg/L metribuzin (Fujifilm Wako). Plants were cultivated under long‐day condition (16‐h light and 8‐h dark) at the indicated photon flux densities.

### Whole genome analysis

Total DNA for whole genome sequencing was extracted from rosette leaves using a DNeasy Plant Pro Kit (QIAGEN, Hilden, Germany) for A251V single mutants and Col‐0 and a Maxwell RSC Plant DNA Kit (Promega) for V219I & A251V double mutants. Paired‐end libraries were prepared with a Nextera Flex DNA Library Prep Kit (Illumina, San Diego, CA) and sequenced on an Illumina NovaSeq 6000 platform by Macrogen Japan, yielding 4 Gb per sample. NGS genome data of Col‐0 were obtained from our previous study (Nakazato *et al*., 2023). For subsequent analyses, we trimmed low‐quality and adaptor sequences from the reads using Platanus_trim v.1.1.0 (http://platanus.bio.titech.ac.jp/pltanus_trim). We then mapped the sequenced reads of each sample to reference sequences (AP000423.1 and BK010421.1) in BWA (v.0.7.12) (Li and Durbin, 2009). We filtered out inadequately mapped reads with mapping identities ≤97% or alignment coverage rates ≤80%. We detected single nucleotide polymorphisms (SNPs) using the bcftools mpileup (‐d 100000 ‐L 100000) and the bcftools call (‐m ‐A) commands (Li *et al*., 2009). Finally, we compiled a list of positions where the difference between the substitution rate of a T_2_ sample and the average substitution rate of the two Col‐0 samples was ≥1%.

### qRT‐PCR analysis

Total RNAs were extracted from one of the two first true leaves 10 days after stratification (DAS) with a Maxwell® RSC Plant RNA Kit (Promega, Madison, WI). qRT‐PCR targeting *petD* and *PEX4* [control gene, AT5G25760 (Czechowski *et al*., 2005)] was performed with One Step TB Green^®^ PrimeScript™ RT‐PCR Kit II (Perfect Real Time) (TaKaRa, Shiga, Japan), using Step One Plus Real Time PCR System (ThermoFisher Scientific). Primers used for this experiment are listed in Table S8.

### Metribuzin resistance test in soil

For the experiments whose results are shown in Figure 2c and Figure S5a,b, seeds were sown on Super Mix A (Sakata Seed Corp., Kanagawa, Japan) in a 200‐cell tray and seedlings were transferred to Y Pot 7.5 cm (Sakata Seed Corp.) filled with Super Mix A at 19 DAS. For the experiments whose results are shown in Figure S5c–f, seeds were sown on culture soil mixed 1:1 with Super Mix A and vermiculite in a 200‐cell tray and seedlings were transferred to Y Pot 7.5 cm filled with this culture soil at 19 DAS. Plants were cultivated under the long‐day condition (16‐h light and 8‐h dark) at a photon flux density of 150 μmol/photons m^2^/s. We sprayed 7.3232 mL of aqueous solution containing 0 or 0.5 g/L of metribuzin on plants on a 23 × 32 cm tray that is shown in Figure 2c and Figure S5 at 25 DAS. Metribuzin was dissolved in dimethyl sulfoxide (DMSO; Fujifilm Wako) and then diluted with sterile distilled water for each concentration. The final concentration of DMSO in the aqueous solution was 0.5% (v/v).

### Measurement of dry weight, leaf area, the number of siliques and chlorophyll content

Plants were cultivated under the long‐day condition (16‐h light and 8‐h dark) at a photon flux density of 150 μmol/photons m^2^/s. For the experiments whose results are shown in Figure 2d–g and Figure S6c–e, seeds were sown on Super Mix A in a 200‐cell tray. At 19 DAS, seedlings were transferred to Jiffy‐7 (plants for silique harvest) or Y Pot 7.5 cm filled with Super Mix A (the other plants). For the experiments whose results are shown in Figure 2h,i and Figure S6a,b, seeds were sown on culture soil mixed 1:1 with Metro‐Mix (Hyponex) and vermiculite. Leaf areas at 25 DAS (Figure 2e, Figure S6c–e) or 4 weeks after sowing (Figure S6b) were measured with Image J. Shoots were harvested at 32 DAS (Figure 2d) or 4 weeks after sowing (Figure S6a), dried at 55 °C and weighed. Roots were harvested 4 weeks after sowing, dried at 55 °C and weighed. Siliques were harvested at 44 DAS, dried at 55 °C and weighed. Four weeks after sowing, SPAD values in the most recently fully expanded leaves were determined with a SPAD‐502 chlorophyll meter (Minolta Camera Co. Ltd., Tokyo, Japan).

### Analysis of chlorophyll fluorescence

Plants were grown on 1/2 MS medium without PPM (See ‘Plant materials for Agrobacterium‐mediated transformation and genotyping’) containing 0 or 1 mg/L metribuzin at 23 °C during a 16‐h light period and 20 °C during an 8‐h dark period under 100 μmol/photons m^2^/s. Chlorophyll fluorescence was analysed with an IMAGING‐PAM chlorophyll fluorescence system (Heinz Walz, Effeltrich, Germany) at 12 DAS. Leaves of the plants that were maintained in darkness overnight were treated with a saturating pulse to obtain maximum fluorescence and then irradiated for over 5 min to obtain steady‐state photosynthesis. Finally, Y(II), Y(NPQ) and Y(NO) were measured at various light intensities following the method of Klughammer and Schreiber (2008).

To examine the chlorophyll fluorescence of plants cultivated under soil conditions, seeds were sown in a 1:1 mixture of Metro‐Mix (Hyponex) and vermiculite. The plants were grown at 22 °C under long‐day conditions (16‐h light, 8‐h dark). Four weeks after sowing, Fv/Fm and Y(II) were measured at various light intensities using an IMAGING‐PAM chlorophyll fluorescence system. Prior to measurement, the plants were irradiated for over 5 min to achieve steady‐state photosynthesis. All plant materials were kept in the dark for at least 30 min before the measurements were taken.

### Modelling the structural conformation of the D1 protein

The docking analysis of metribuzin (PubChem CID 30479) with the wild‐type D1 protein of PSII was carried out by using AutoDock Vina 1.1.2 (Trott and Olson, 2010) and UCSF Chimera v.1.16 (Pettersen *et al*., 2004). This analysis focused on the Q_B_ site. The best pose of metribuzin binding to the site is shown in Figure 4c,d. The binding energy of metribuzin was −5.8 kcal/mol. The structure of the wild‐type D1 protein was referenced from a previous study (Graça *et al*., 2021) (PDB code: 7OUI). Structures of the D1 protein of A251V mutants and V219I & A251V double mutants were predicted using ColabFold v.1.5.3 (AlphaFold2 using MMseqs2) (Jumper *et al*., 2021; Mirdita *et al*., 2022). Figure 4 is prepared using PyMOL v.2.5.0 (Schrödinger, LLC).

### Statistical analysis

The significance of differences was measured with Tukey–Kramer multiple comparison tests by using Microsoft Excel for Fv/Fm and Y(II) (Figure 3a,b) and R Studio for the remaining figures.

## Conflict of interest

A patent related to the method described in this paper is pending in Japan and USA. The patent is held by the University of Tokyo.

## Author contributions

I.N., N.T. and S.A. designed the study. I.N., W.Y., H.M. and S.A. wrote the paper. I.N. generated transformants, performed genotyping and metribuzin resistance tests, measured dry weights and leaf areas and analysed *psbA* sequences. W.Y. and Y.Q. measured and analysed the dry weights, leaf areas, SPAD and photosynthetic activity. H.M. modelled the 3D structure of the D1 protein. M.O. analysed the NGS data.

## Supporting information


**Figure S1** 219th and 251st amino acids in the D1 protein of crops.
**Figure S2** Introduction of V219I and A251V mutations in the D1 protein encoded by *psbA*.
**Figure S3** Growth of *psbA* mutants in the presence of 1 mg/L metribuzin.
**Figure S4** Sequencing the chloroplast genome of *psbA* mutants.
**Figure S5** Metribuzin treatment on *psbA* mutants in soil.
**Figure S6** Shoot dry weights and leaf areas of *psbA* mutants in soil.
**Figure S7** Photosynthetic characteristics of *psbA* mutants in the absence or presence of metribuzin.
**Figure S8** Photosynthetic characteristics of *psbA* mutants cultivated in soil conditions without metribuzin.

**Table S1** Sequence of the D1 protein.
**Table S2** Genotypes of T_1_ plants of *psbA*655‐1 and *psbA*655_2.
**Table S3** Genotypes of T_1_ plants of *psbA*752‐6_v2.
**Table S4** Genotypes of T_2_ plants of *psbA*655‐1 and *psbA*752‐6_v2.
**Table S5** T_1_ and T_2_ plants whose progenies were subjected to metribuzin resistance test and other phenotyping assays.
**Table S6** Base editing frequency of T_1_ plants of *psbA*752‐6_v2 (V219I mutants background).
**Table S7** The sequences that the TALE domains bind to.
**Table S8** Primer sequences used in this study.

## Data Availability

All data are available in this paper and supporting information. Plasmids were deposited in Addgene.
