# Supplementary material for: Resistance to the herbicide metribuzin conferred to Arabidopsis thaliana by targeted base editing of the chloroplast genome
Source: Plant Biotechnol J. 2024 Oct 20;23(1):204–15. doi: 10.1111/pbi.14490 (PMC11672748; doi:10.1111/pbi.14490)
Supplement: Supplementary file 1 — Figure S1 219th and 251st amino acids in the D1 protein of crops. Figure S2 Introduction of V219I and A251V mutations in the D1 protein encoded by psbA. Figure S3 Growth of psbA mutants in the presence of 1 mg/L metribuzin. Figure S4 Sequencing the chloroplast genome of psbA mutants. Figure S5 Metribuzin treatment on psbA mutants in soil. Figure S6 Shoot dry weights and leaf areas of psbA mutants in soil. Figure S7 Photosynthetic characteristics of psbA mutants in the absence or presence of metribuzin. Figure S8 Photosynthetic characteristics of psbA mutants cultivated in soil conditions without metribuzin. [file PBI-23-204-s002.docx]

| Scientific name (Reference sequence) | Amino acid | | DNA sequence around the codon of | |
| --- | --- | --- | --- | --- |
|  | 219^th^ | 251^st^ | V219 (TALE binding sequences are underlined) | A251 (TALE binding sequences are underlined) |
| *Arabidopsis thaliana*  (NC_000932.1) | Val | Ala | codon for V219  TTTAGTGCTATGCATGGTTCCTTGGTAACTTCTAGTTTGATCAGGGAAACCA | codon for A251  CAAGAAGAAGAAACTTACAACATTGTAGCTGCTCACGGTTATTTTGGCCGATTGA |
| *Brassica oleracea var. italica*  (MN649876.1) | Val | Ala | TTTAGTGCTATGCATGGTTCTTTGGTAACTTCTAGTTTGATCAGGGAAACCA | CAAGAAGAAGAAACTTACAACATTGTAGCTGCTCACGGTTATTTTGGCCGATTGA |
| *Brassica rapa subsp. pekinensis*  (NC_015139.1) | Val | Ala | TTTAGTGCTATGCATGGTTCTTTGGTAACTTCTAGTTTGATCAGGGAAACCA | CAAGAAGAAGAAACTTACAACATTGTAGCTGCTCACGGTTATTTTGGCCGATTGA |
| *Brassica napus*  (GQ861354.1) | Val | Ala | TTTAGTGCTATGCATGGTTCTTTGGTAACTTCTAGTTTGATCAGGGAAACCA | CAAGAAGAAGAAACTTACAACATTGTAGCTGCTCACGGTTATTTTGGCCGATTGA |
| *Raphanus sativus*  (NC_024469) | Val | Ala | TTTAGTGCTATGCATGGTTCTTTGGTAACTTCTAGTTTGATCAGGGAAACCA | CAAGAAGAAGAAACTTACAACATTGTAGCTGCTCACGGTTATTTTGGCCGATTGA |
| *Glycine max*  (NC_007942.1) | Val | Ala | TTCAGTGCTATGCATGGTTCCTTGGTAACTTCTAGTTTGATCAGGGAAACCA | CAAGAGGAAGAAACCTATAATATTGTAGCTGCTCATGGTTATTTTGGCCGATTGA |
| *Cucumis sativus*  (NC_007144.1) | Val | Ala | TTCAGTGCTATGCATGGTTCCTTGGTAACTTCTAAGTTGATCAGGGAAACCA | CAAGAGGAAGAAACTTATAATATCGTAGCTGCTCATGGTTATTTTGGCCGATTGA |
| *Solanum melongena*  (KU682719.1) | Val | Ala | TTCAGTGCTATGCATGGTTCCTTGGTAACTTCTAGTTTGATCAGGGAAACCA | CAAGAGGAAGAAACTTATAATATCGTAGCTGCTCATGGTTATTTTGGCCGATTGA |
| *Lactuca sativa*  (NC_007578.1) | Val | Ala | TTTAGTGCTATGCATGGTTCTTTGGTAACCTCTAGTTTGATCAGGGAAACCA | CAAGAAGAAGAAACTTATAATATCGTAGCCGCTCATGGTTATTTTGGCCGATTGA |
| *Allium cepa*  (NC_024813.1) | Val | Ala | TTTAGTGCTATGCATGGTTCCTTGGTAACCTCTAGTTTAATCAGGGAAACTA | CAAGAGGAAGAAACTTATAATATCGTTGCTGCTCATGGTTATTTTGGCCGATTGA |
| *Oryza sativa*  (NC_001320.1) | Val | Ala | TTCAGTGCTATGCATGGTTCCTTGGTAACCTCTAGTTTGATCAGGGAAACCA | CAAGAGGAAGAAACTTATAATATTGTGGCCGCTCATGGTTATTTTGGCCGATTAA |
| *Sorghum bicolor*  (NC_008602.1) | Val | Ala | TTCAGTGCTATGCATGGTTCCTTGGTAACCTCTAGTTTGATCAGGGAAACCA | CAGGAAGAAGAGACTTATAATATTGTGGCTGCTCACGGTTATTTCGGTCGATTAA |
| *Zea mays*  (NC_001666.2) | Val | Ala | TTCAGTGCTATGCATGGTTCCCTTGTAACCTCTAGTTTGATCAGGGAAACCA | CAGGAAGAAGAGACTTATAATATTGTGGCTGCTCACGGTTATTTTGGTCGATTAA |

Variant nucleotides compared to the reference (*A. thaliana* ) are shown in red. The nucleotides responsible for V219I and A251V substitutions are shown in blue.


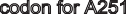

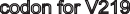


**Figure S1 219^th^ and 251^st^ amino acids in the D1 protein of crops.** 219^th^ and 251^st^ amino acids in crops and the nucleotide sequences around the codons for these two amino acids are shown. Amino acid sequences of the D1 protein from 211^th^ to 275^th^ amino acids in 275 species of photosynthetic organisms are shown in Table S1.

**a**

***psbA*655-1 (*psbA*2-1)**

N-terminal domain

C-terminal domain

**CD half UGI**

**CD-half combination**

(Left - Right)

1. **1333N**-**1333C**
2. **1397C**-**1397N**
3. **1397N**-**1397C**

**5′-...ATTTTCTGTGGTTTCCCTGATCAAACT AGAAGTTACCAAGGAACCATGCATAGCACTAAA AAGGGAGC...-3′**

**platinum TALE left**

**9**

**3′-...TAAAAGACACCAAAGGGACTAGTTTGA TGTTCAATGGTTCCTTGGTACGTATCGTGATTT TTCCCTCG...-5′**

**platinum TALE right**

: Target window

**UGI**

**CD half**

C-terminal

N-terminal

**G/C**: Special targets predicted to confer metribuzin resistance


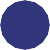

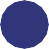


domain

domain

**b** Target: 655th G (V219I) 11 DAS

| **Construct**  **name** | **CD half**  **(Left-Right)** | **Type** | ***Position in the target window*** | | | | | | | | | | | | | | | **Nos. of**  **T_1_ plants** |
| --- | --- | --- | --- | --- | --- | --- | --- | --- | --- | --- | --- | --- | --- | --- | --- | --- | --- | --- |
| ***psbA* 655-1**  **(*psbA* 2-1)** | **1397N-1397C** |  |  | *1* | *2* | *3* | *4* | *5* | *6* | *7* | *8* | *9* | *10* | *11* | *12* | *13* | *14* | 19 |
|  |  | h/c |  |  | 1 |  |  | 12 |  |  |  | 8 | 7 |  |  |  |  |  |
|  |  | homo |  |  |  |  |  | 3 |  |  |  | 9 | 10 |  |  |  |  |  |
| ***psbA* 655-2** | **1397N-1397C** |  | *1* | *2* | *3* | *4* | *5* | *6* | *7* | *8* | *9* | *10* | *11* | *12* | *13* | *14* |  | 6 |
|  |  | h/c |  |  | 3 |  |  | 4 |  |  |  |  |  |  |  |  |  |  |
|  |  | homo |  |  | 2 |  |  | 2 |  |  |  | 6 | 6 |  |  |  |  |  |

5'- T A G A A G T T A C C A A G G -3'

| T | C | T | T | C | A | A | T | G | G | T | T |
| --- | --- | --- | --- | --- | --- | --- | --- | --- | --- | --- | --- |

I

| coding strand 3'- A | | |  | |  | | | C C -5' | | |
| --- | --- | --- | --- | --- | --- | --- | --- | --- | --- | --- |
| ↓ | | | ↓ | | ↓ | | | ↓ | | |
| T | T | T | T | T | A | **A T A** | A | T | T |  |
| S221F | | | T220 | | ***V219I***  ***Special target*** | | | L218L | | |

**c** Target: 752nd C (A251V)

**ptpTALECD**

| **Construct**  **name** | **CD half**  **(Left-Right)** | **Type** | ***Position in the target window*** | | | | | | | | | | | | | | | | | | **Nos. of**  **T_1_ plants** |
| --- | --- | --- | --- | --- | --- | --- | --- | --- | --- | --- | --- | --- | --- | --- | --- | --- | --- | --- | --- | --- | --- |
| ***psbA***  **752-1** | **1333N-1333C** |  |  | | *1* | *2* | *3* | *4* | *5* | *6* | *7* | *8* | *9* | *10* | *11* | *12* | *13* | *14* | *15* | *16* | 9 |
|  |  | h/c |  |  |  |  |  |  |  |  |  |  |  |  |  |  |  |  |  |  |  |
|  |  | homo |  |  |  |  |  |  |  |  |  |  |  |  |  |  |  |  |  |  |  |
| ***psbA***  **752-2** | **1333N-1333C** |  |  |  | *1* | *2* | *3* | *4* | *5* | *6* | *7* | *8* | *9* | *10* | *11* | *12* | *13* | *14* | *15* | *16* | 4 |
|  |  | h/c |  |  |  |  |  |  |  |  |  |  | 2 |  | 2 |  |  |  |  |  |  |
|  |  | homo |  |  |  |  |  |  |  |  |  |  |  |  | 1 |  |  |  |  |  |  |
| ***psbA***  **752-3** | **1333N-1333C** |  |  | *1* | *2* | *3* | *4* | *5* | *6* | *7* | *8* | *9* | *10* | *11* | *12* | *13* | *14* | *15* | *16* |  | 9 |
|  |  | h/c |  |  |  |  |  |  |  |  |  |  |  |  | 3 |  |  |  |  |  |  |
|  |  | homo |  |  |  |  |  |  |  |  |  |  |  |  |  |  |  |  |  |  |  |
|  | **1397C-1397N** |  |  | *1* | *2* | *3* | *4* | *5* | *6* | *7* | *8* | *9* | *10* | *11* | *12* | *13* | *14* | *15* | *16* |  | 8 |
|  |  | h/c |  |  |  |  |  |  |  |  |  |  |  |  | 1 |  |  |  |  |  |  |
|  |  | homo |  |  |  |  |  |  |  |  |  |  |  |  | 6 |  |  |  |  |  |  |
| ***psbA***  **752-4** | **1333N-1333C** |  | *1* | *2* | *3* | *4* | *5* | *6* | *7* | *8* | *9* | *10* | *11* | *12* | *13* | *14* | *15* | *16* |  | | 6 |
|  |  | h/c |  |  |  |  |  |  |  |  |  |  |  |  |  |  |  |  | | |  |
|  |  | homo |  |  |  |  |  |  |  |  |  |  |  |  |  |  |  |  |  |  |  |
|  | **1397C-1397N** |  | *1* | *2* | *3* | *4* | *5* | *6* | *7* | *8* | *9* | *10* | *11* | *12* | *13* | *14* | *15* | *16* |  | | 8 |
|  |  | h/c |  |  |  |  |  |  |  |  |  |  |  |  |  |  |  |  | | |  |
|  |  | homo |  |  |  |  |  |  |  |  |  |  |  |  | 7 |  |  |  |  |  |  |
| ***psbA***  **752-5_v1** | **1397N-1397C** |  |  | *1* | *2* | *3* | *4* | *5* | *6* | *7* | *8* | *9* | *10* | *11* | *12* |  | | | | | 16 |
|  |  | h/c |  |  |  |  |  |  |  |  |  |  |  |  |  |  |  |  |  |  |  |
|  |  | homo |  |  |  |  |  |  |  |  |  |  |  |  |  |  |  |  |  |  |  |
| ***psbA***  **752-6_v1** | **1397N-1397C** |  |  | *1* | *2* | *3* | *4* | *5* | *6* | *7* | *8* | *9* | *10* | *11* | *12* | *13* | *14* |  | | | 12 |
|  |  | h/c |  |  |  |  |  |  |  |  |  |  |  |  |  |  |  |  |  |  |  |
|  |  | homo |  |  |  |  |  |  |  |  |  |  |  |  | 7 |  |  |  |  |  |  |
| coding strand 5'- A T T G T A G C T | | | | | | | | | | | | G C T | | | C A C | | | G G T -3' | | | |

↓ ↓

T A C

**G T T**

***A251V***

***Special target***

H252Y

| **Construct**  **name** | **CD half**  **(Left-Right)** | **Type** | ***Position in the target window*** | | | | | | | | | | | | | | | **Nos. of**  **T_1_ plants** |
| --- | --- | --- | --- | --- | --- | --- | --- | --- | --- | --- | --- | --- | --- | --- | --- | --- | --- | --- |
| ***psbA***  **752-5_v2** | **1397C-1397N** |  |  | | *1* | *2* | *3* | *4* | *5* | *6* | *7* | *8* | *9* | *10* | *11* | *12* |  | 16 |
|  |  | h/c |  |  |  |  |  |  |  | 1 | 1 |  |  |  |  |  |  |  |
|  |  | homo |  |  |  |  |  |  |  |  |  |  |  |  |  |  |  |  |
| ***psbA***  **752-6_v2** | **1397C-1397N** |  | *1* | *2* | *3* | *4* | *5* | *6* | *7* | *8* | *9* | *10* | *11* | *12* | *13* | *14* |  | 29 |
|  |  | h/c |  |  | 21 |  | 12 |  |  | 14 |  |  |  |  |  |  |  |  |
|  |  | homo |  |  | 2 |  | 13 |  |  | 1 |  |  |  |  |  |  |  |  |
| ***psbA***  **752-7** | **1397C-1397N** |  |  | *1* | *2* | *3* | *4* | *5* | *6* | *7* | *8* | *9* | *10* | *11* | *12* | *13* | *14* | 16 |
|  |  | h/c |  |  |  |  |  |  |  |  |  |  |  |  |  |  |  |  |
|  |  | homo |  |  |  |  |  |  |  |  |  |  |  |  |  |  |  |  |
| ***psbA***  **752-8** | **1397C-1397N** |  |  | *1* | *2* | *3* | *4* | *5* | *6* | *7* | *8* | *9* | *10* | *11* | *12* | *13* |  | 15 |
|  |  | h/c |  |  | 1 |  | 1 |  |  |  | 1 |  |  |  |  |  |  |  |
|  |  | homo |  |  |  |  |  |  |  |  |  |  |  |  |  |  |  |  |

5'- G T G A G C A G C T A C A A T -3'

**ptpTALECD_v2**

| C | A | C | T | C | G | T | C | G |
| --- | --- | --- | --- | --- | --- | --- | --- | --- |

coding strand 3'-

↓

A T G T T A -5'

↓ ↓

| C A T | **T T G** | T | C | A |
| --- | --- | --- | --- | --- |
| H252Y ***A251V*** | | T | T | G |
| ***Special target*** | | T | T | A |

A250V A250T A250I


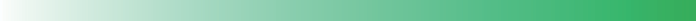


0 The number of edited plants/ The number of T_1_ plants (%)

100

**Figure S2 Introduction of V219I and A251V mutations in the D1 protein encoded by *psbA*.** (a**)** ptpTALECD or ptpTALECD_v2 and its target sequence. Platinum TALE is a site-specific DNA- binding protein. CD, cytidine deaminase. UGI, uracil glycosylase inhibitor. CD was split at Gly 1333 or Gly 1397 and N- and C-terminal halves were fused to the left or right platinum TALE domains, respectively. For example, in 1333N-1333C, CD was split at Gly 1333 and N- and C-terminal halves were fused to left and right platinum TALE domains, respectively. Modified from Nakazato *et al*., 2021. (b, c**)** Targeted base editing in *psbA*. Numbers of T_1_ plants that had edited bases at each position in the target windows. Predicted amino acid substitutions are also shown. Editing efficiencies are shown by the intensity of the green color. h/c, heteroplasmically and/or chimerically substituted; homo homoplasmically substituted. Targets of b and c were 655^th^ G (V219I) and 752^nd^ C (A251V), respectively. The data for the plants with ptpTALECD targeting *psbA*655-1 were derived from Nakazato *et al*., 2023.

**a** 1/2 MS plate

1/2 MS plate (Metribuzin free)

(containing 1 mg/L of Metribuzin)


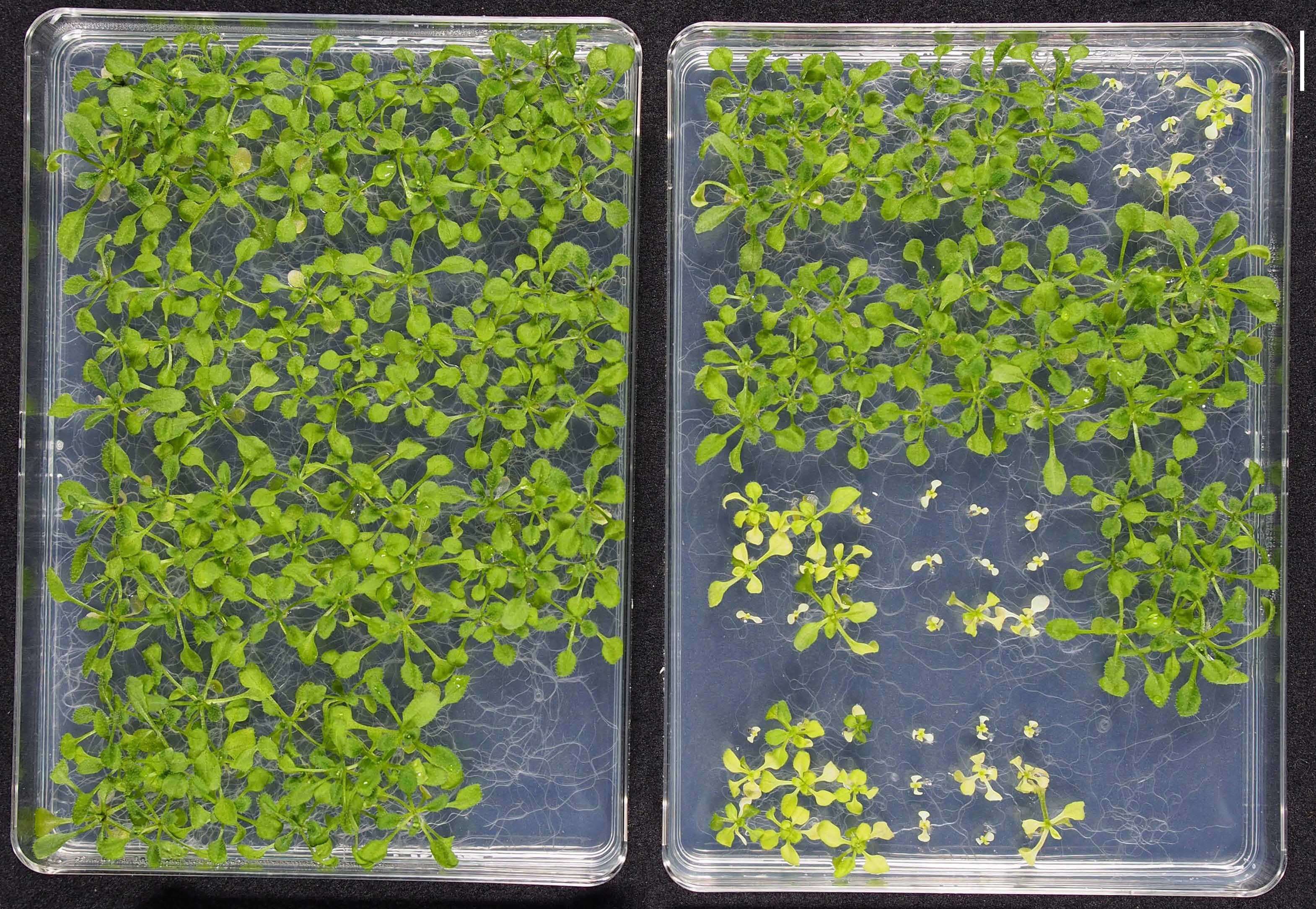


The light intensity was 75 μmol photons m-2 s-1

**b**

| **A251V**  **Line2** | **A250T & A251V**  **Line 2** | **Col-0** |
| --- | --- | --- |
| **A251V**  **Line1** | **A250T & A251V**  **Line 1** | **V219I & A251V**  **Line 2*** |
| **V219I**  **Line 2** | **A250T**  **Line 2** | **V219I & A251V**  **Line 1*** |
| **V219I**  **Line 1** | **A250T**  **Line 1** |  |

*T_2_ plants

The other mutants were in the T_3_ generation.

**Figure S3 Growth of *psbA* mutants in the presence of 1 mg L^-1^ metribuzin.** (a**)** Growth of *psbA* mutants at 19 days after stratification on 1/2 MS agar plates containing 0 or 1 mg L^-1^ of metribuzin. Nine seeds were sown on one plate for each line. These plants were cultivated under a photon flux density of 75 μmol photons m^-2^ s^-1^. We did not check the genotypes of T_2_ plants of V219I & A251V double mutants, but these plants are supposed to inherit V219I & A251V double mutants. This is because their siblings stably inherited the V219I & A251V double mutations (Table S4). Bar = 1 cm.

(b**)** The map of the plate shown in (a). This plate is divided into 12 regions, each containing a mutant or a wild-type plant.

**a**

| SNPs information | | | | | Allele Frequency | | | | | | | |
| --- | --- | --- | --- | --- | --- | --- | --- | --- | --- | --- | --- | --- |
| Position (bp) | Gene | Annotation | REF | ALT | Average of two Col-0 plants | V219I & A251V | | | A251V | | | |
|  |  |  |  |  |  | Line 3 | Line 4 | Line 5 | Line 1 | Line 2 | Line 3 | Line 4 |
| 693 | *psbA* | 752^nd^ C (A251V) | G | A | 0.0 | 99.8 | 99.8 | 99.7 | 99.7 | 99.8 | 99.7 | 99.8 |
| 790 |  | 655^th^ G (V219I) | C | T | 0.0 | 100.0 | 100.0 | 100.0 | 0.0 | 0.0 | 0.0 | 0.0 |
| 791 |  | 654^th^ G (L218L) | C | T | 0.0 | 100.0 | 100.0 | 100.0 | 0.0 | 0.0 | 0.0 | 0.0 |
| 77,185 | *petD* | intron | C | T | 0.0 | 0.0 | 0.0 | 0.0 | 99.9 | 0.0 | 0.0 | 0.0 |

**Target mutation**

**Bystander error Off-target**


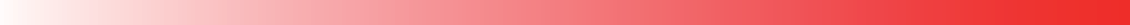


0 Mutant allele frequency (%) 100

**b**

1.5

a

a

b

Relative accumlation level of mature *petD* mRNA

1.0

0.5

0.0

Genotype

Col-0

A251V with an off-target mutation in the *petD* intron A251V

**Figure S4 Sequencing the chloroplast genome of *psbA* mutants.** (a**)** On- and off-target mutations in the chloroplast genome of seven T2 plants, whose progenies were subjected to metribuzin-resistant assay and showed metribuzin resistance. Detected C:G-to-T:A mutations, where allele frequencies differed from the reference genome (AP000423.1) by more than 1% in one or more plants, are listed. Mutant allele frequencies are shown by the intensity of the red color. Read depth varied from 4,891 to 10,385. (b**)** Accumulation of mature *petD* mRNAs between wild-type plants, A251V mutants (A251V Line 2, T3 generation), and A251V mutants with an off-target mutation in the *petD* intron (A251V Line 1, T_3_ generation). The relative values of the amount of *petD* mRNA standardized by that of *PEX4* mRNA are shown (wild-type plants as 1). Different letters indicate significantly different values (P < 0.05, Tukey-Kramer multiple comparison test). Error bars represent standard deviation. *n* (the number of plants) = 5.

1. **0 g L-1 metribuzin (control) 0.5 g L-1 metribuzin**


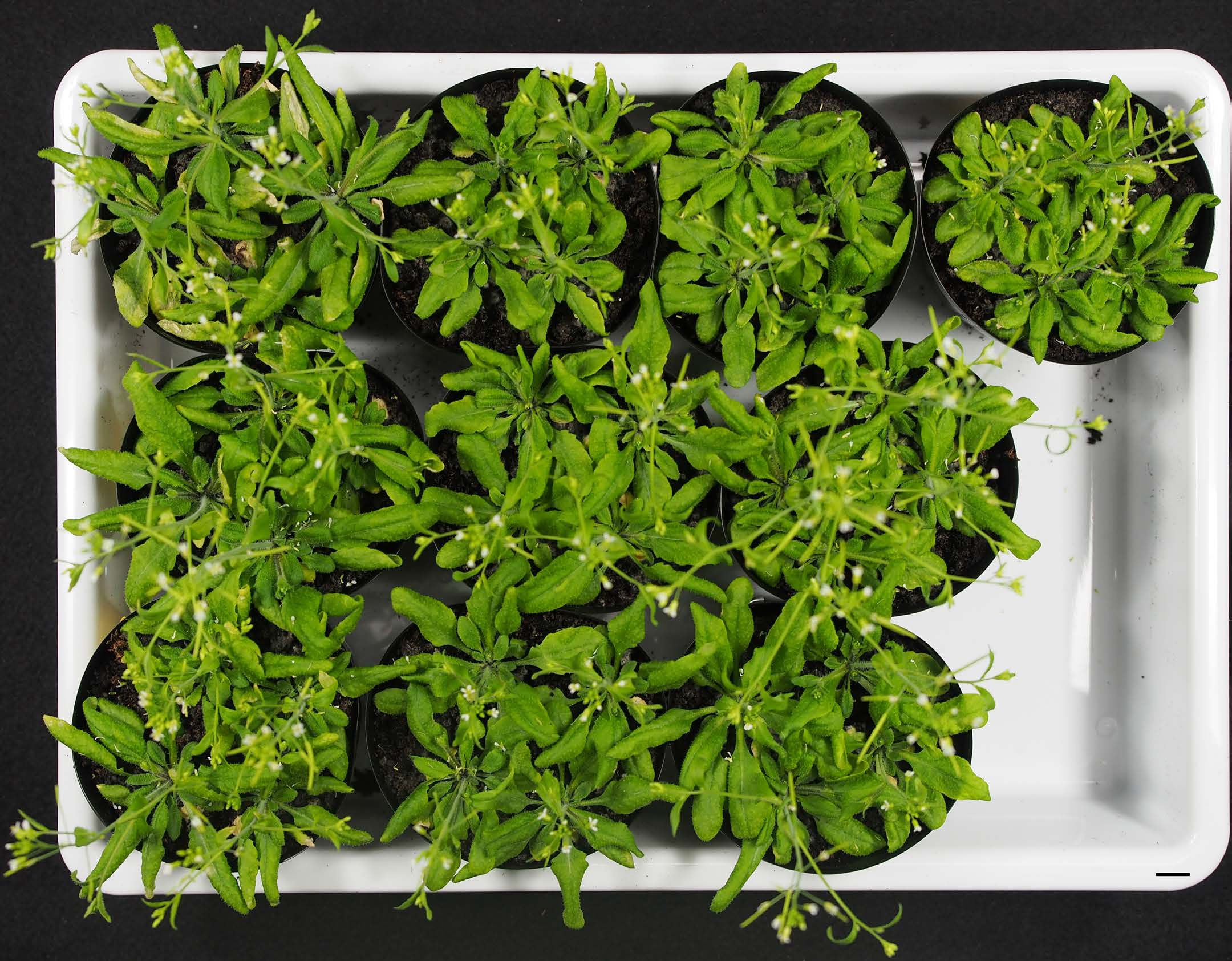

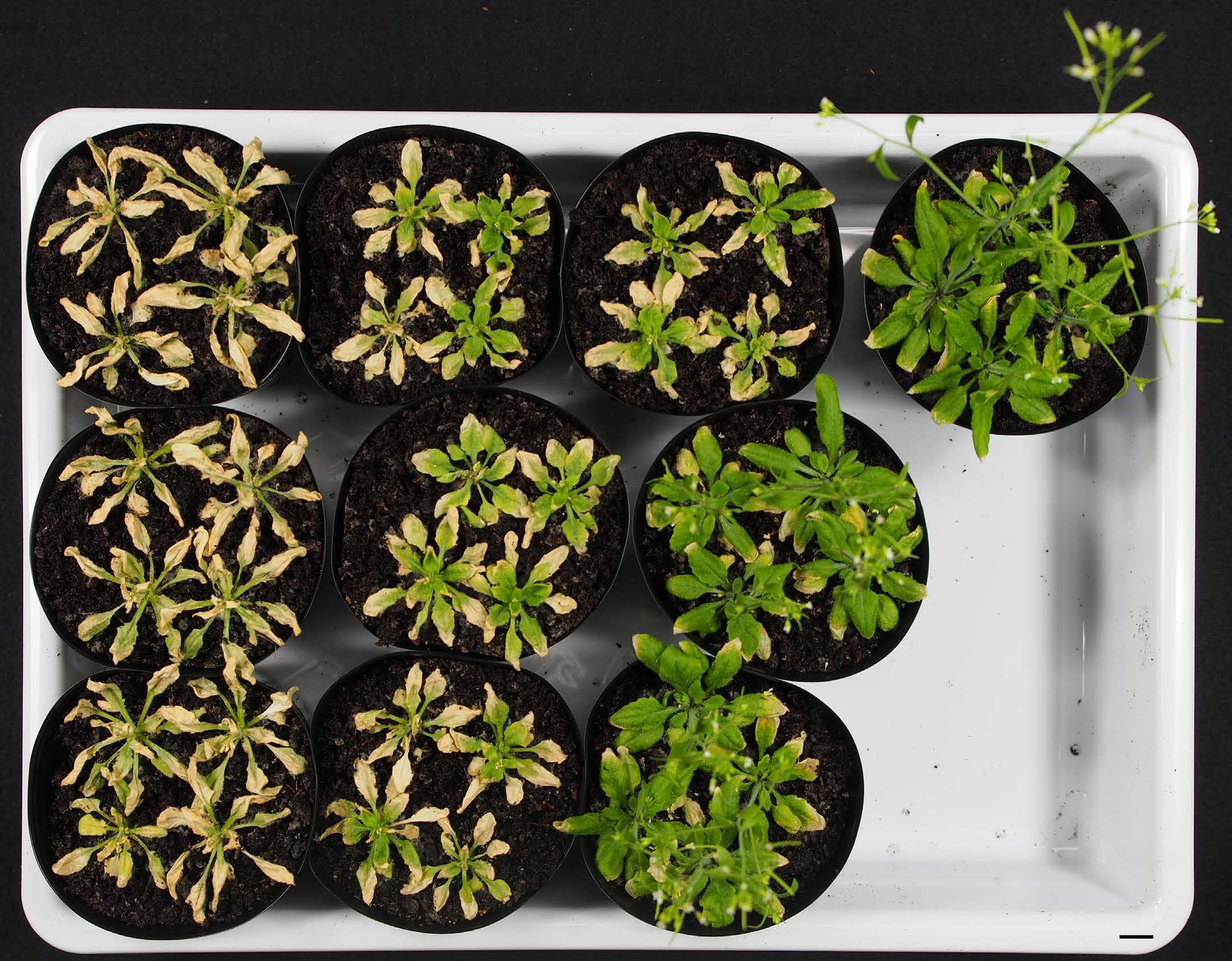

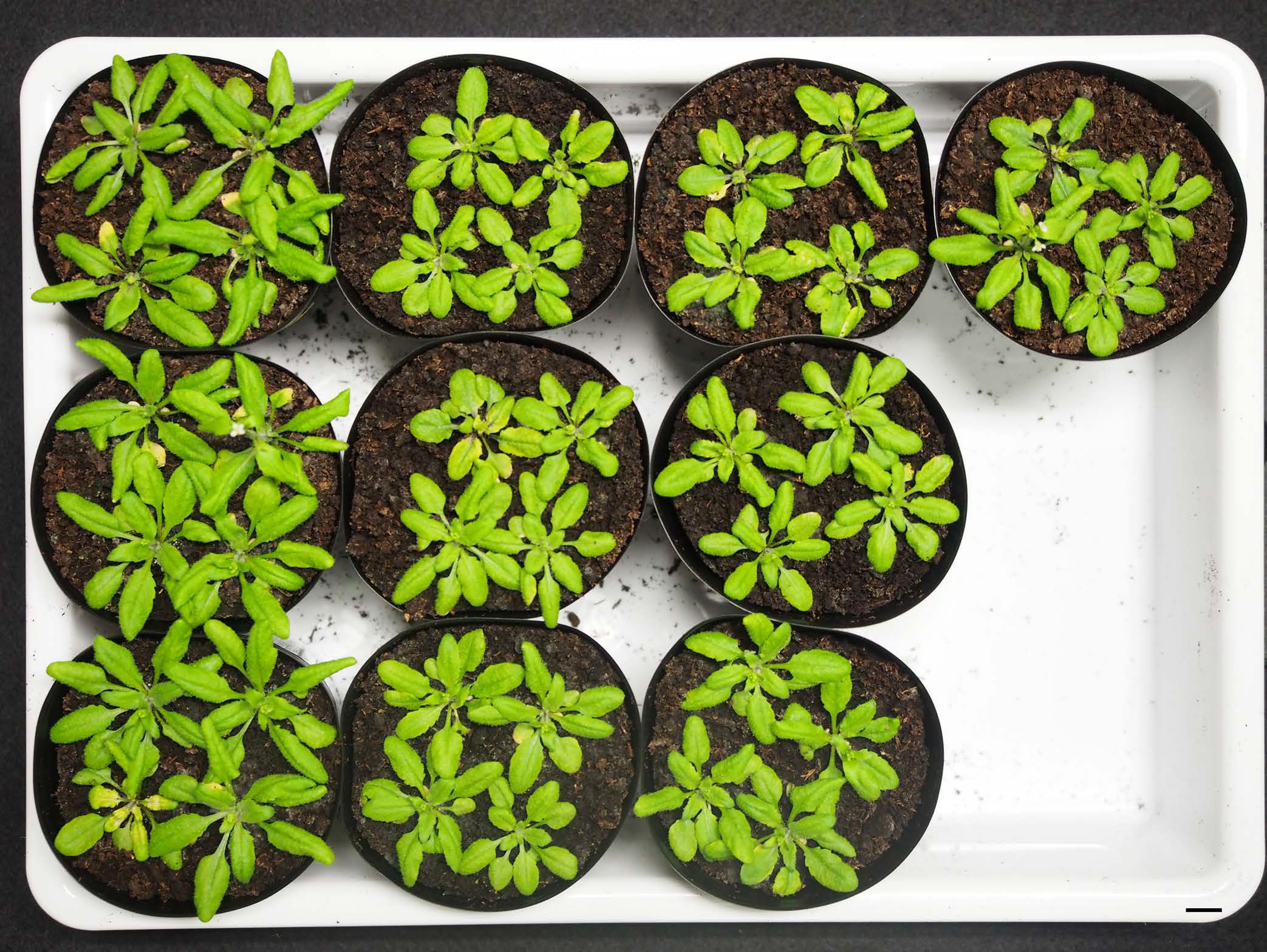

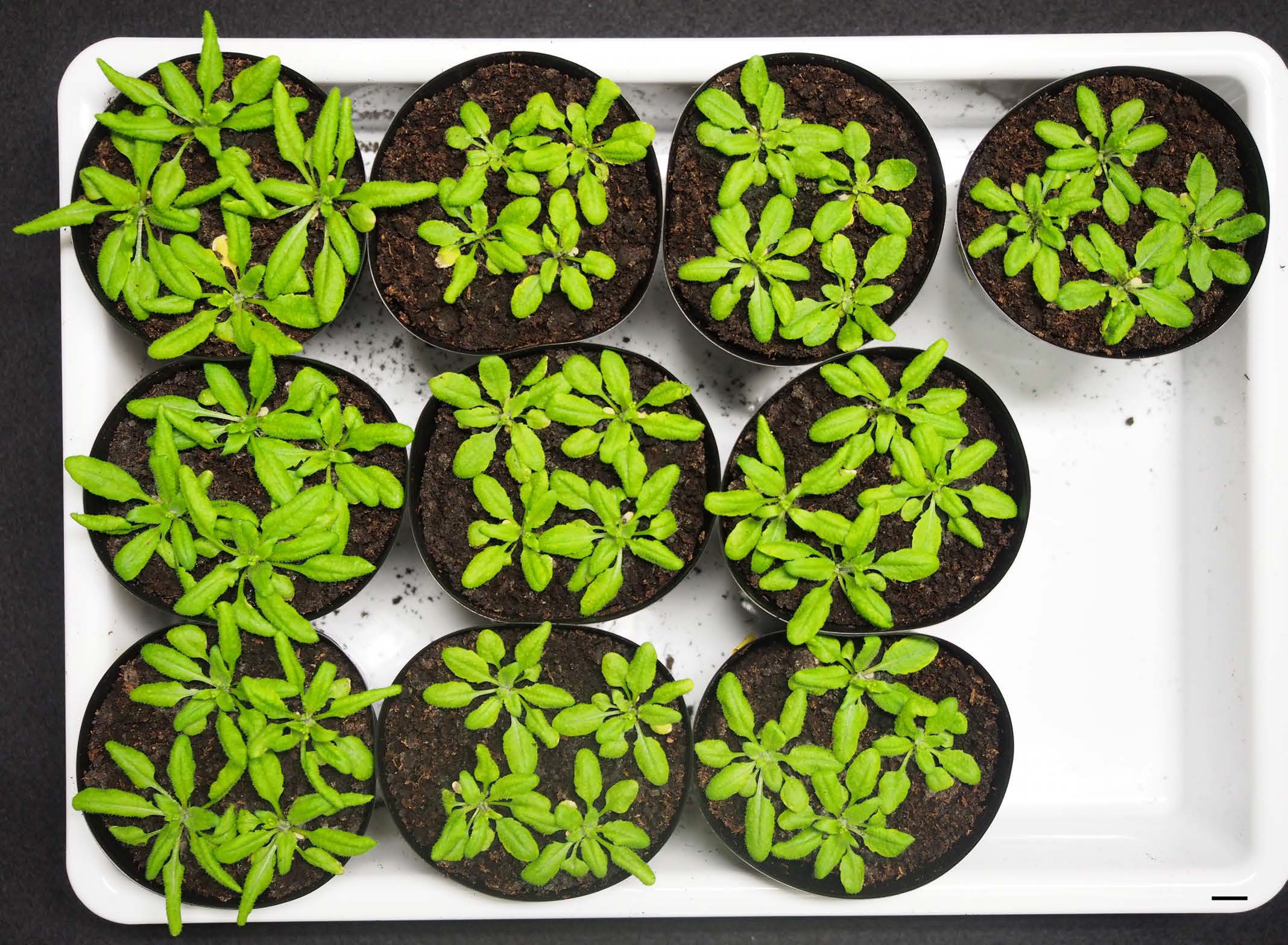


Experimental Replicate 2

**6 days after metribuzin treatment**

**Before metribuzin treatment**

**c 0 g L-1 metribuzin (control) 0.5 g L-1 metribuzin**

Experimental Replicate4

**8 days after metribuzin treatment**

**Before metribuzin treatment**


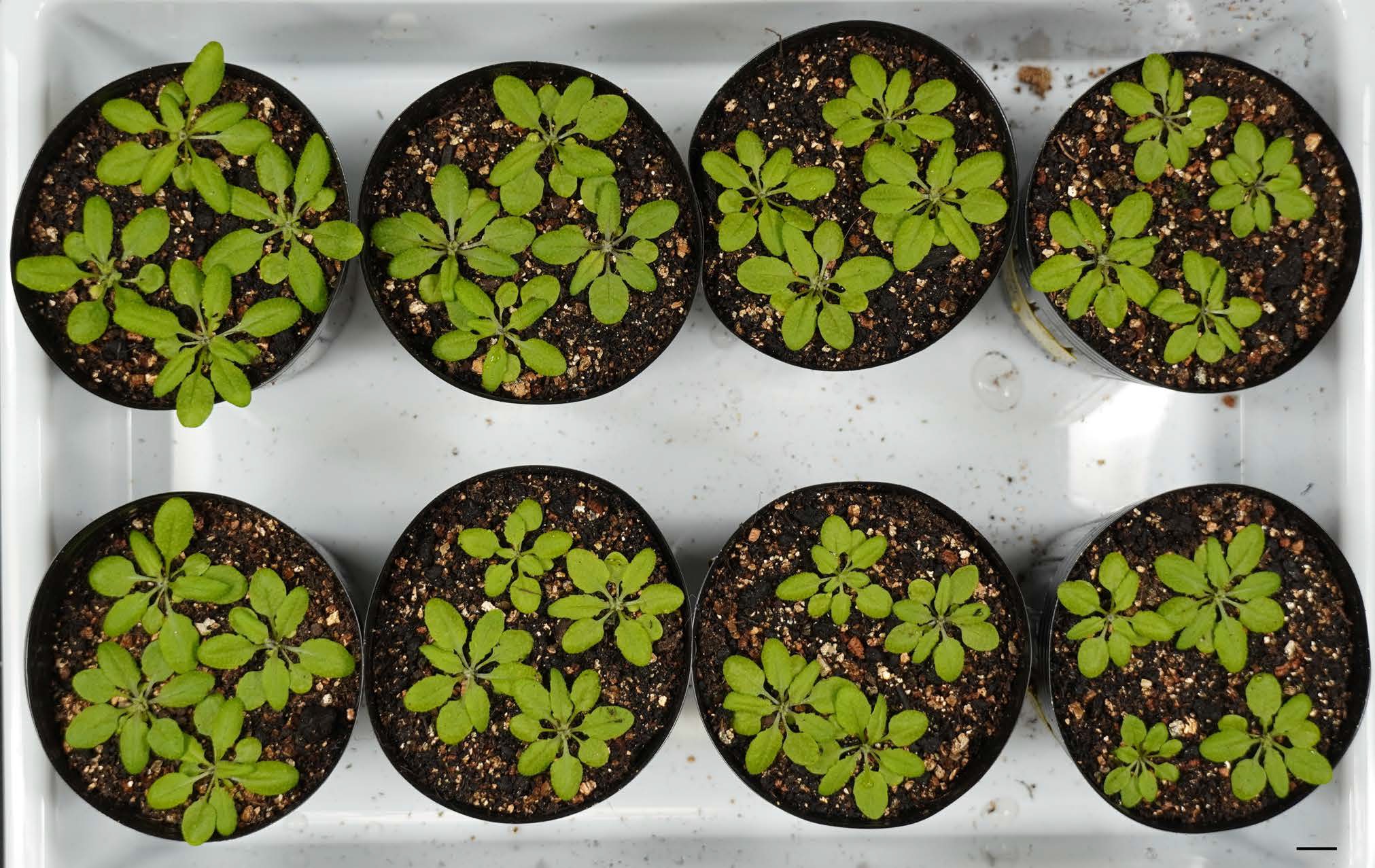

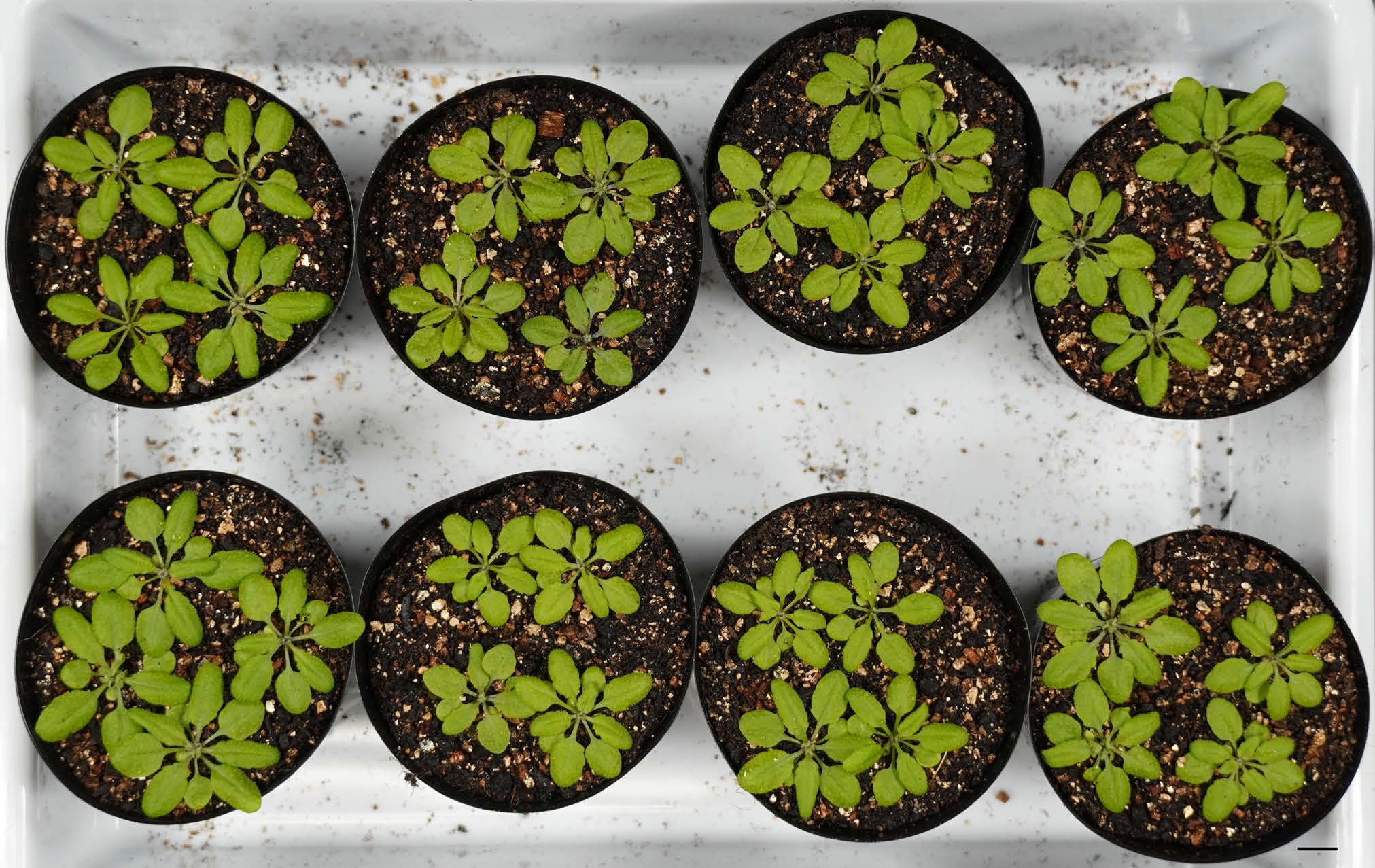


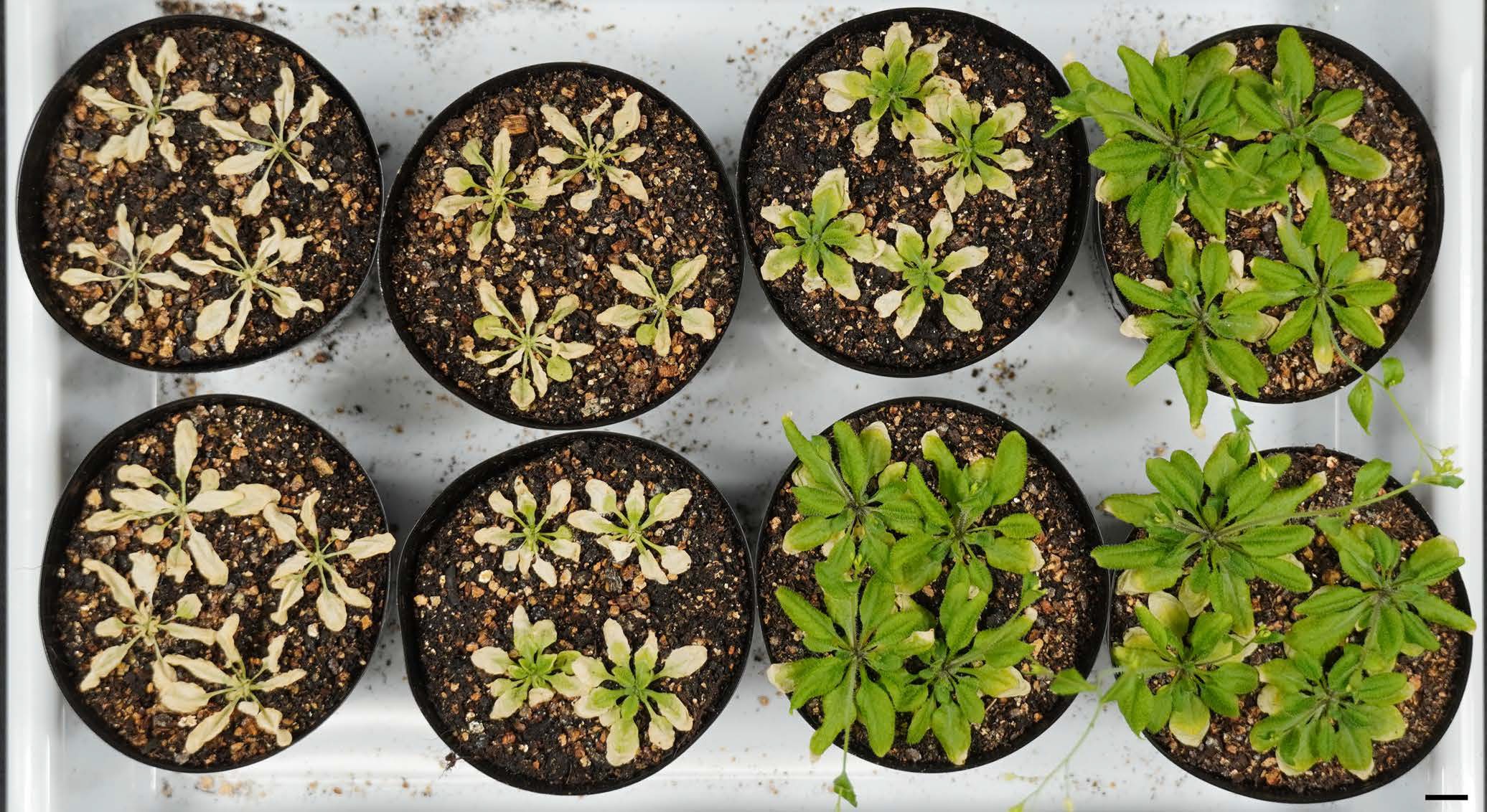


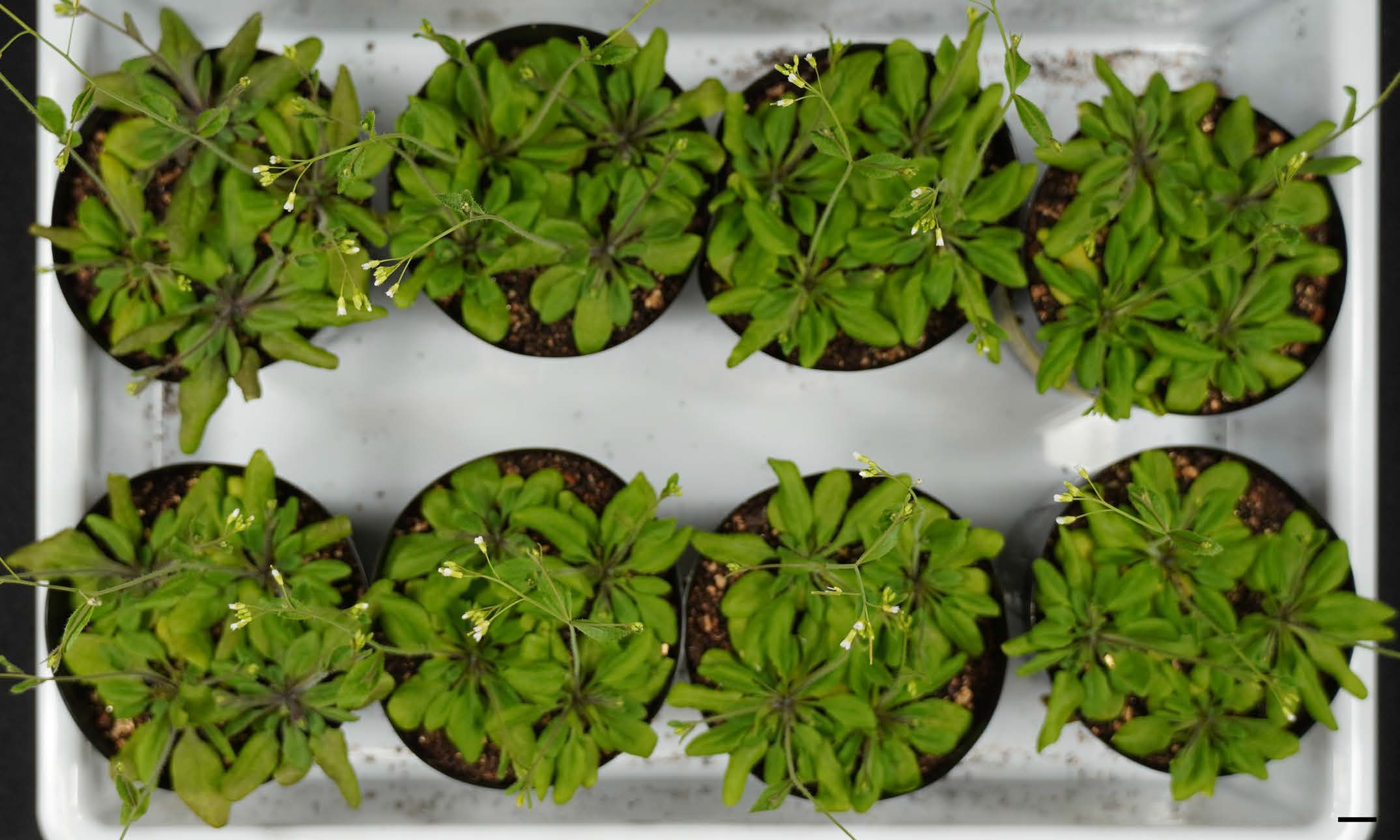
**e 0 g L-1 metribuzin (control) 0.5 g L-1 metribuzin**

Experimental Replicate 6

**Before metribuzin treatment**

Experimental Replicate 7

1. **0 g L-1 metribuzin (control) 0.5 g L-1 metribuzin**


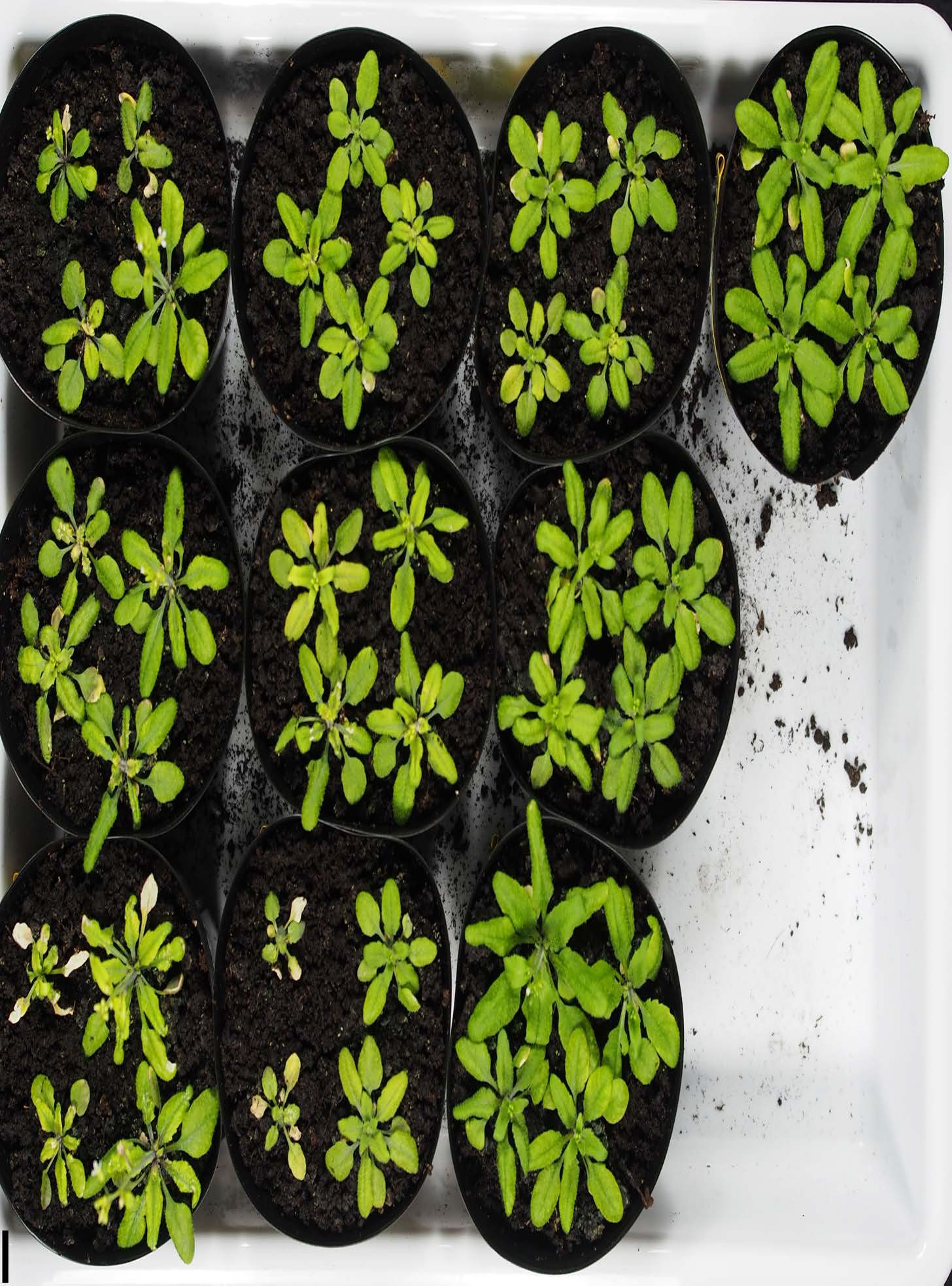

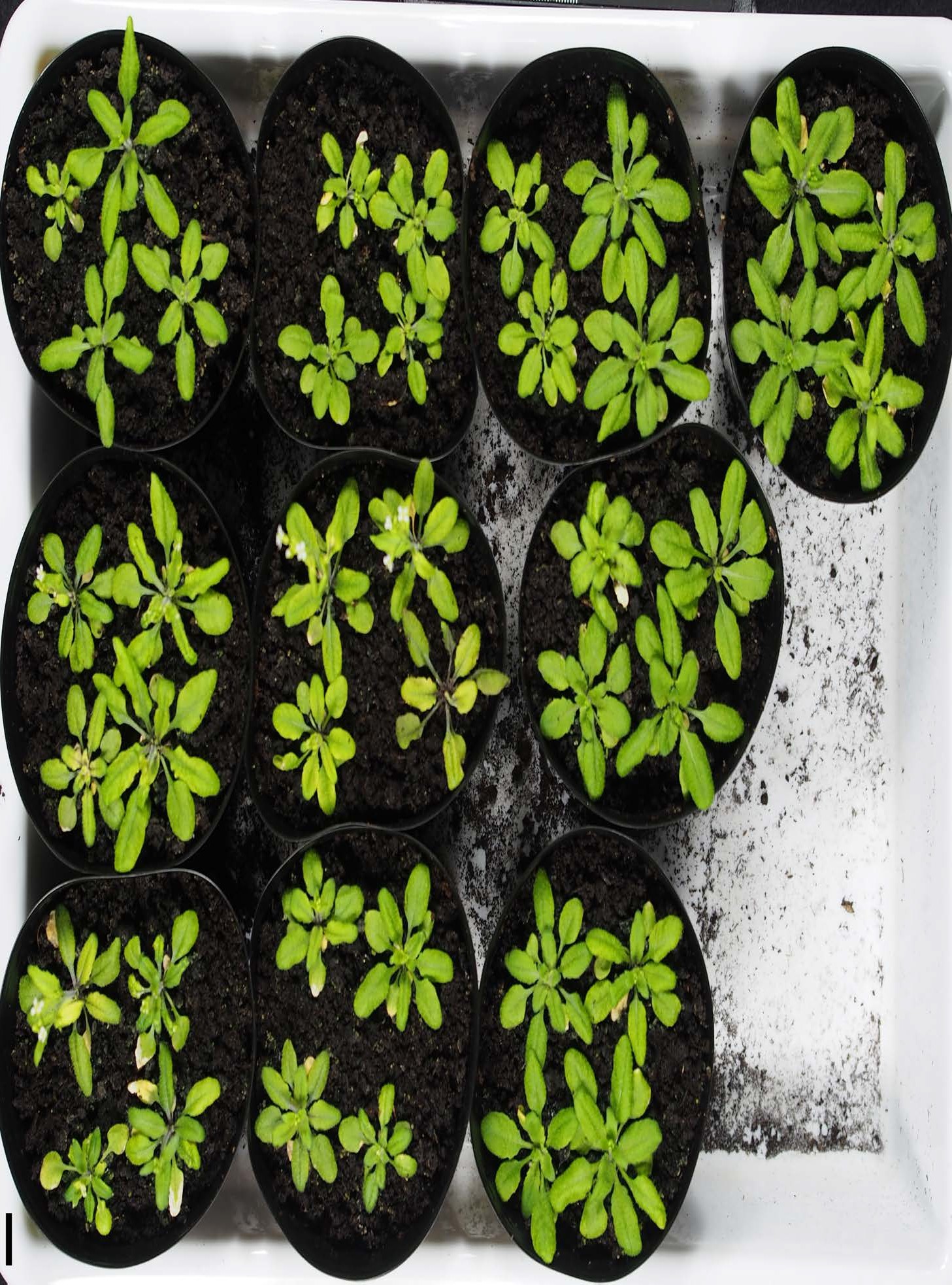


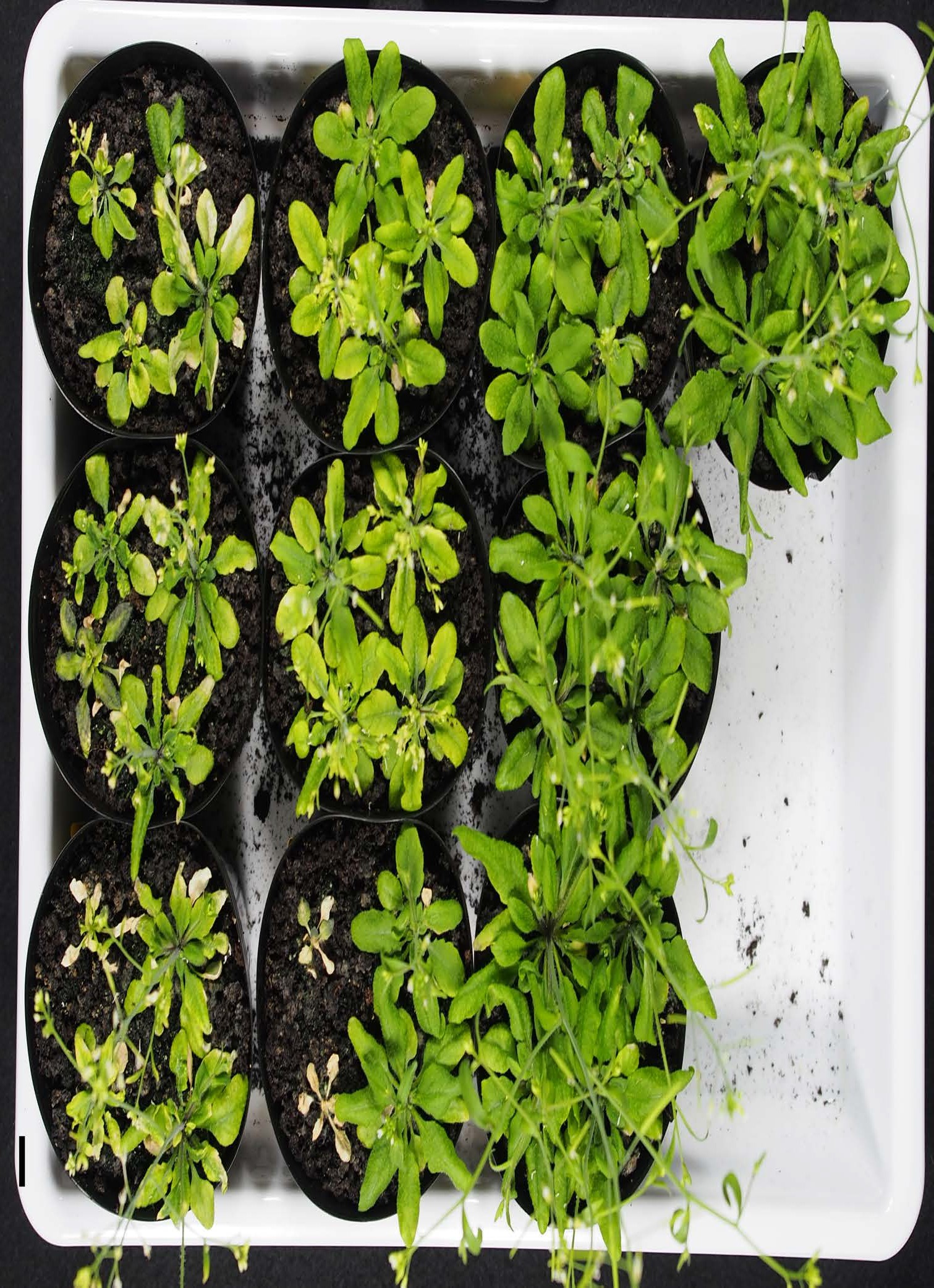


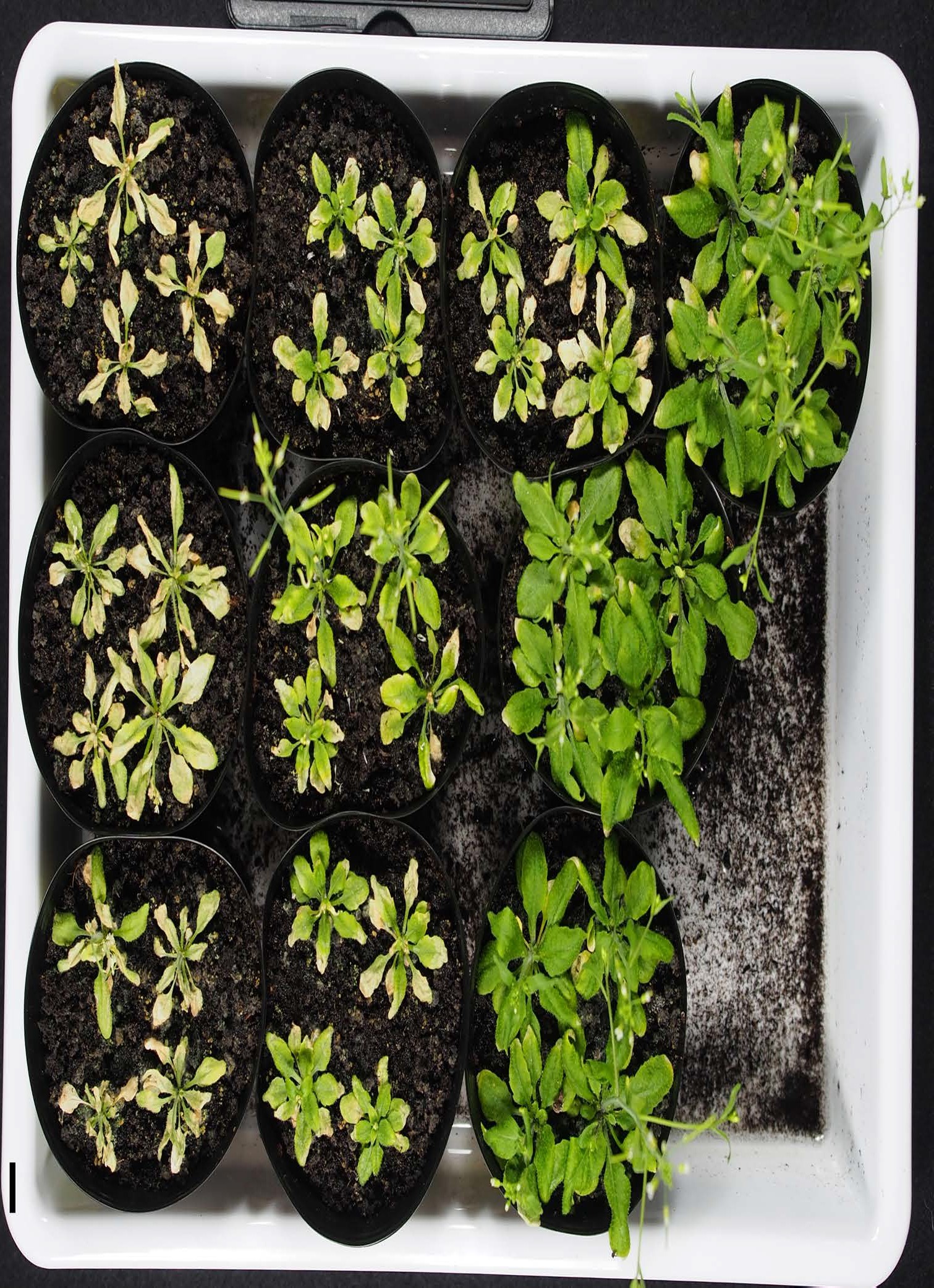
**d 0 g L-1 metribuzin (control) 0.5 g L-1 metribuzin**

Experimental Replicate 5

Experimental Replicate 3

**8 days after metribuzin treatment**

**Before metribuzin treatment**

**6 days after metribuzin treatment**

**Before metribuzin treatment**


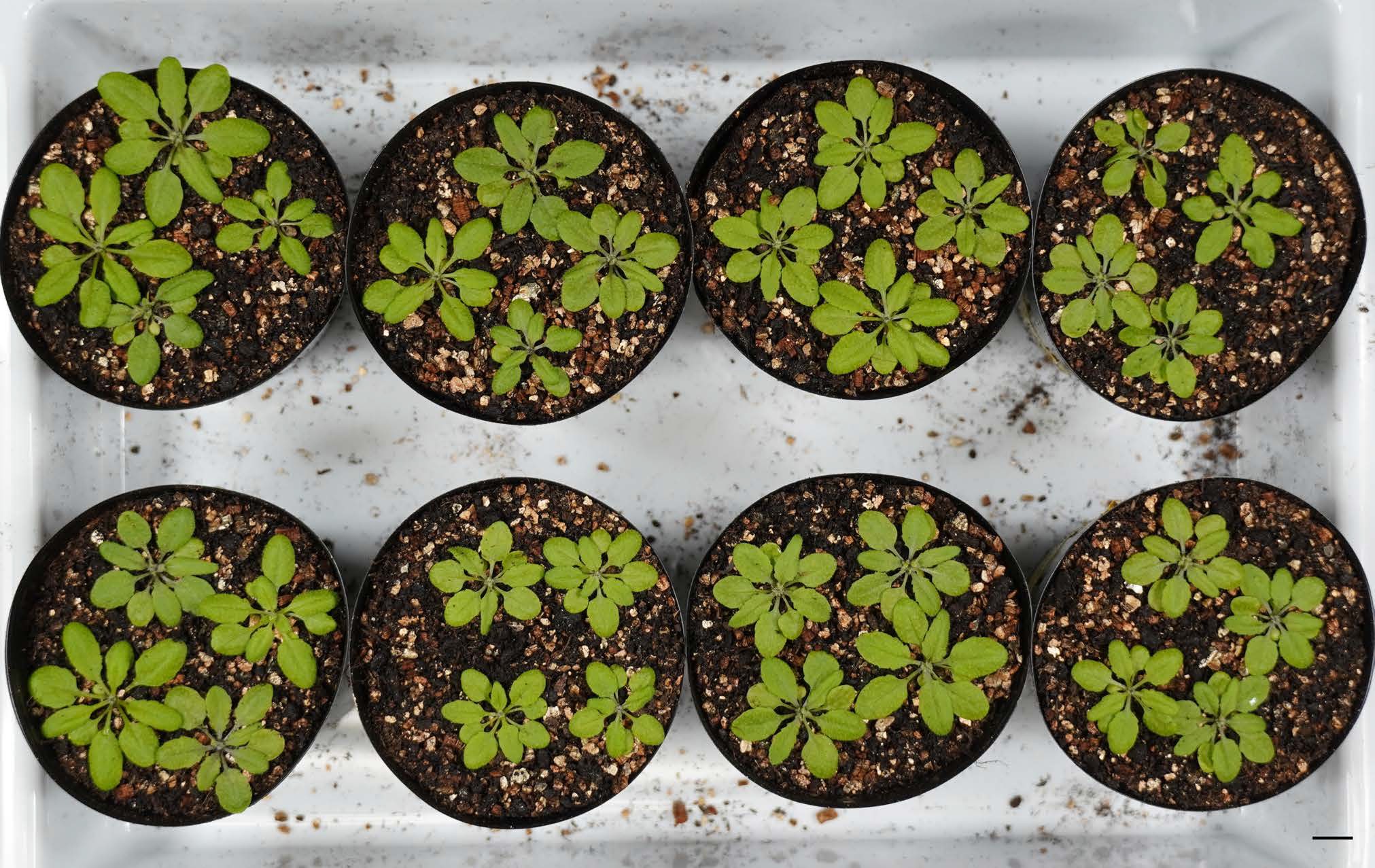

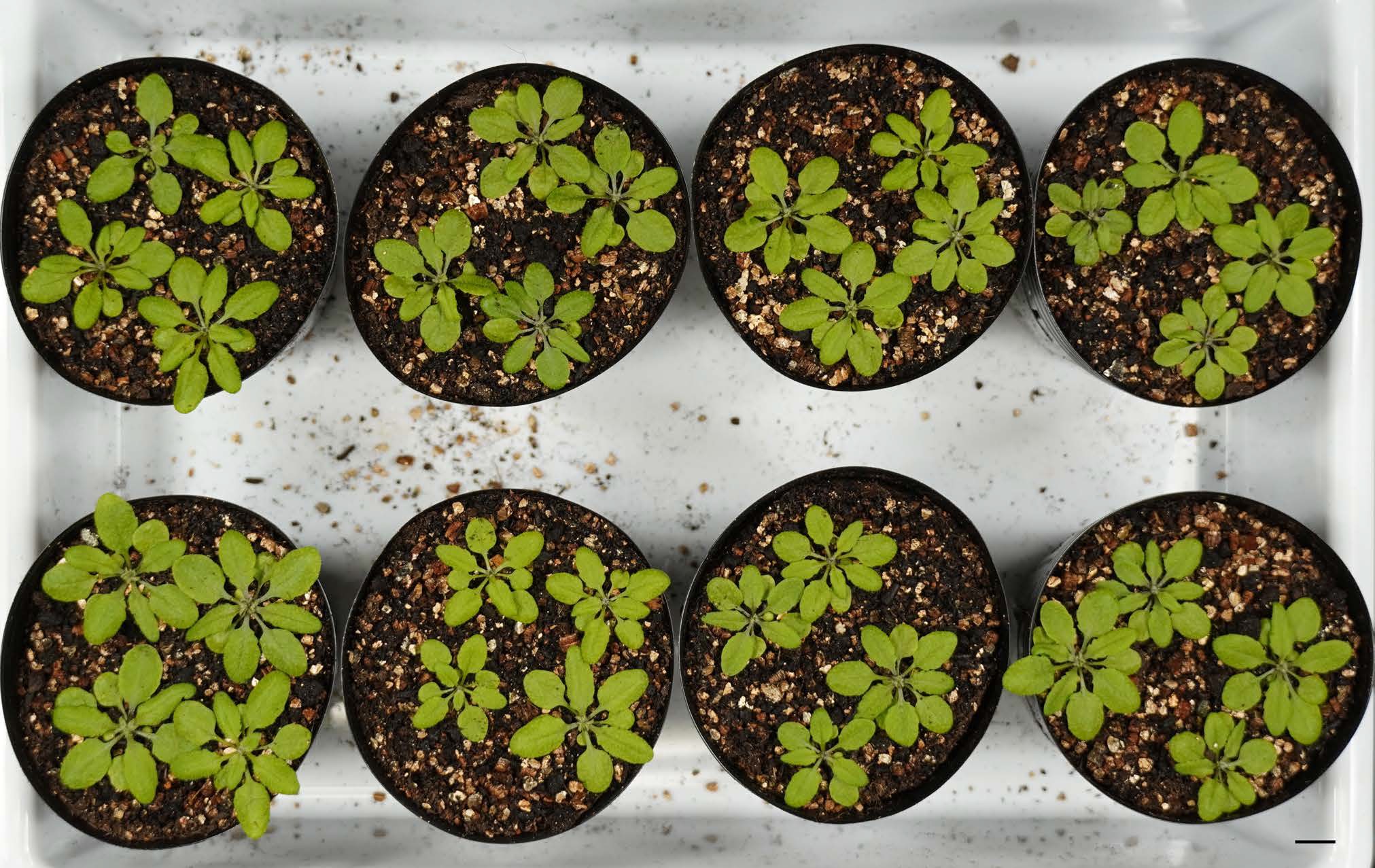


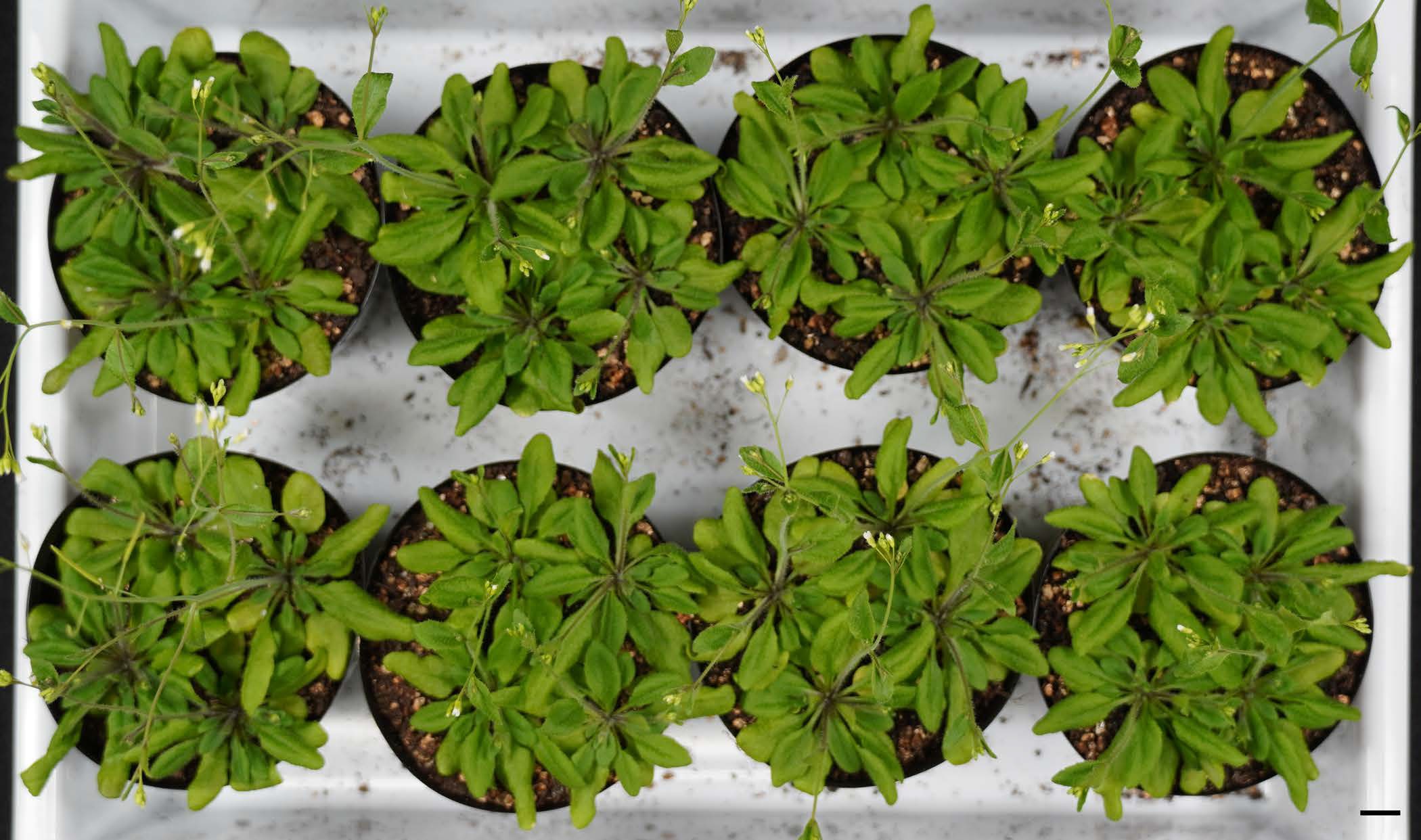

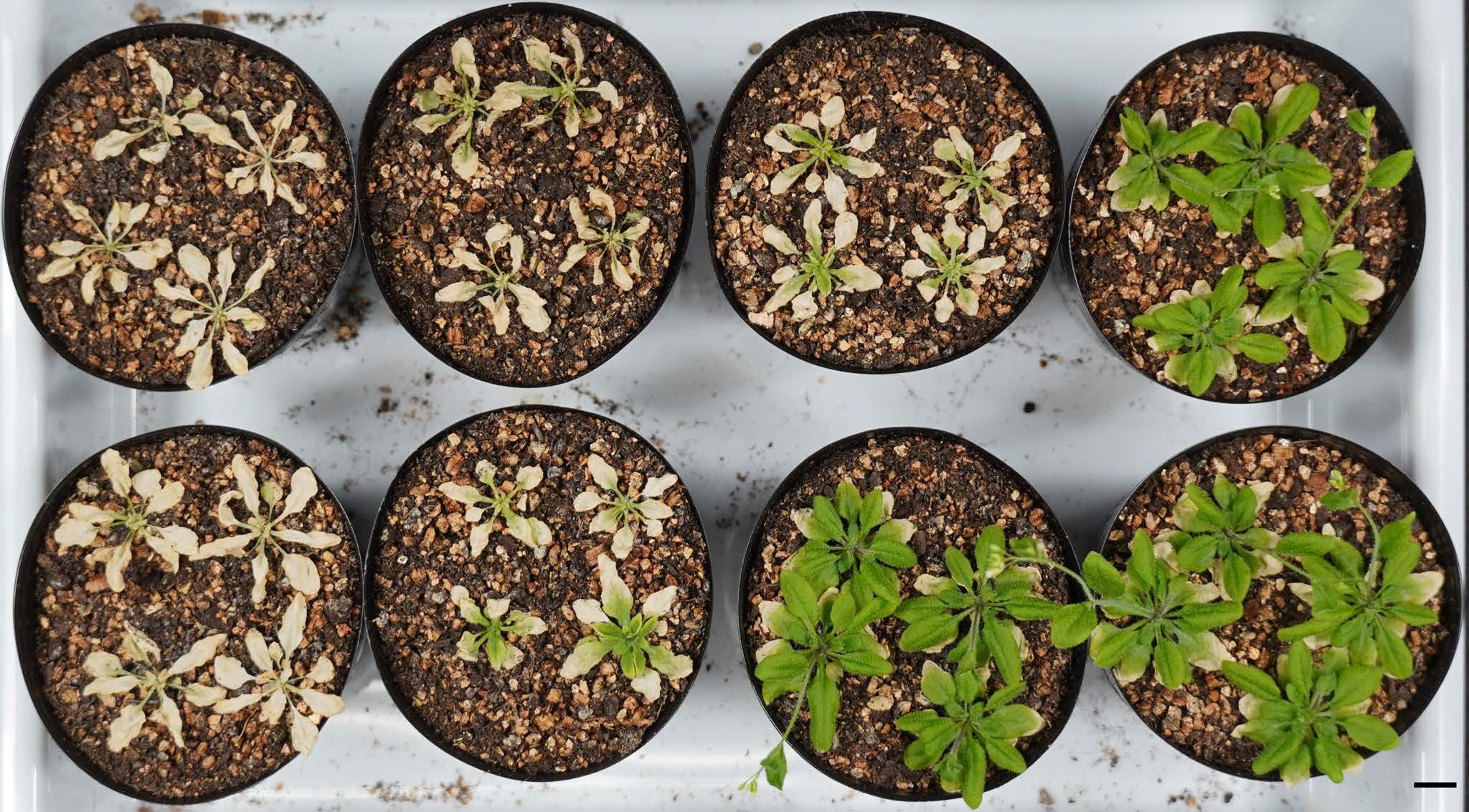


**f 0 g L-1 metribuzin (control) 0.5 g L-1 metribuzin**

**Before metribuzin treatment**


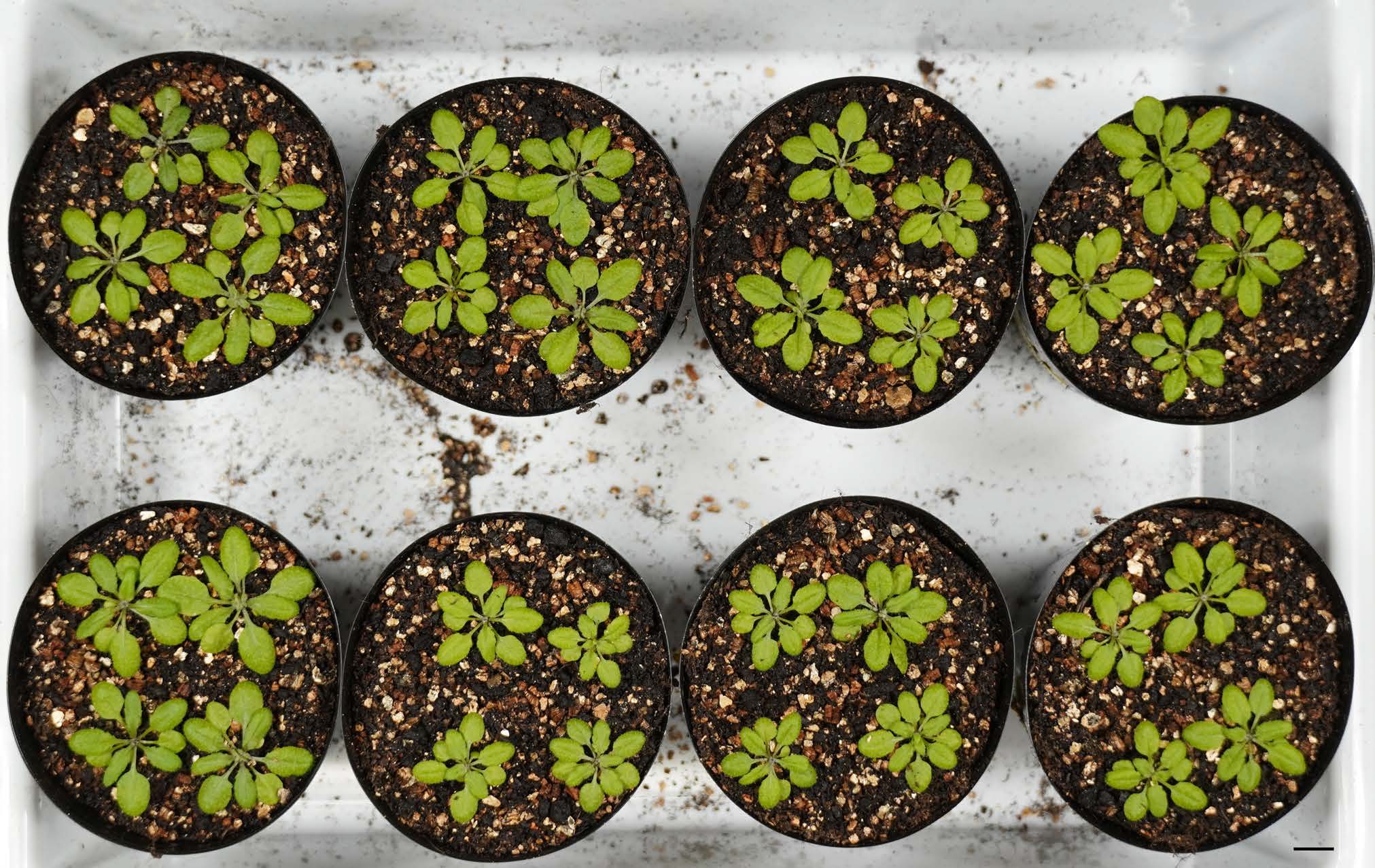

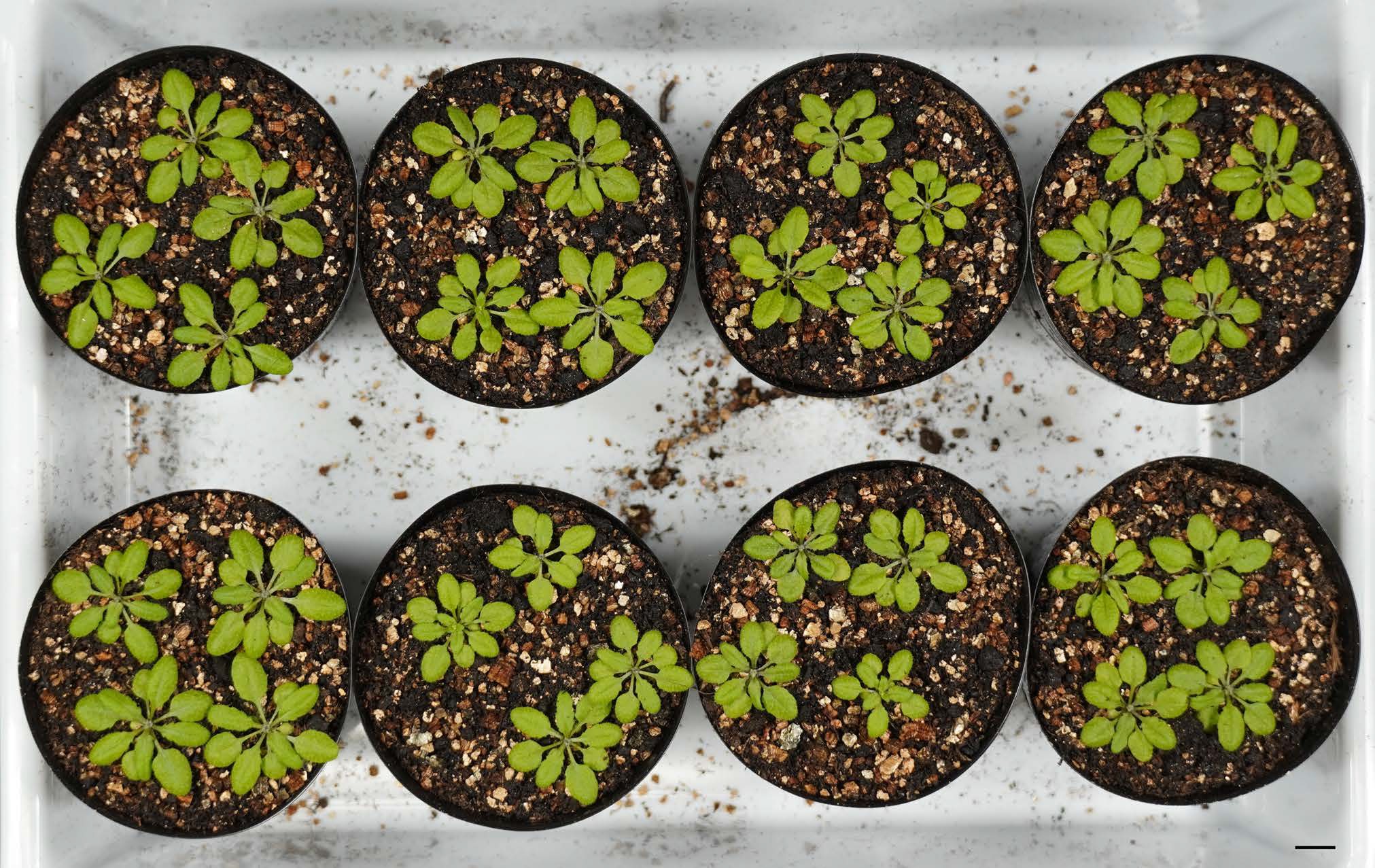

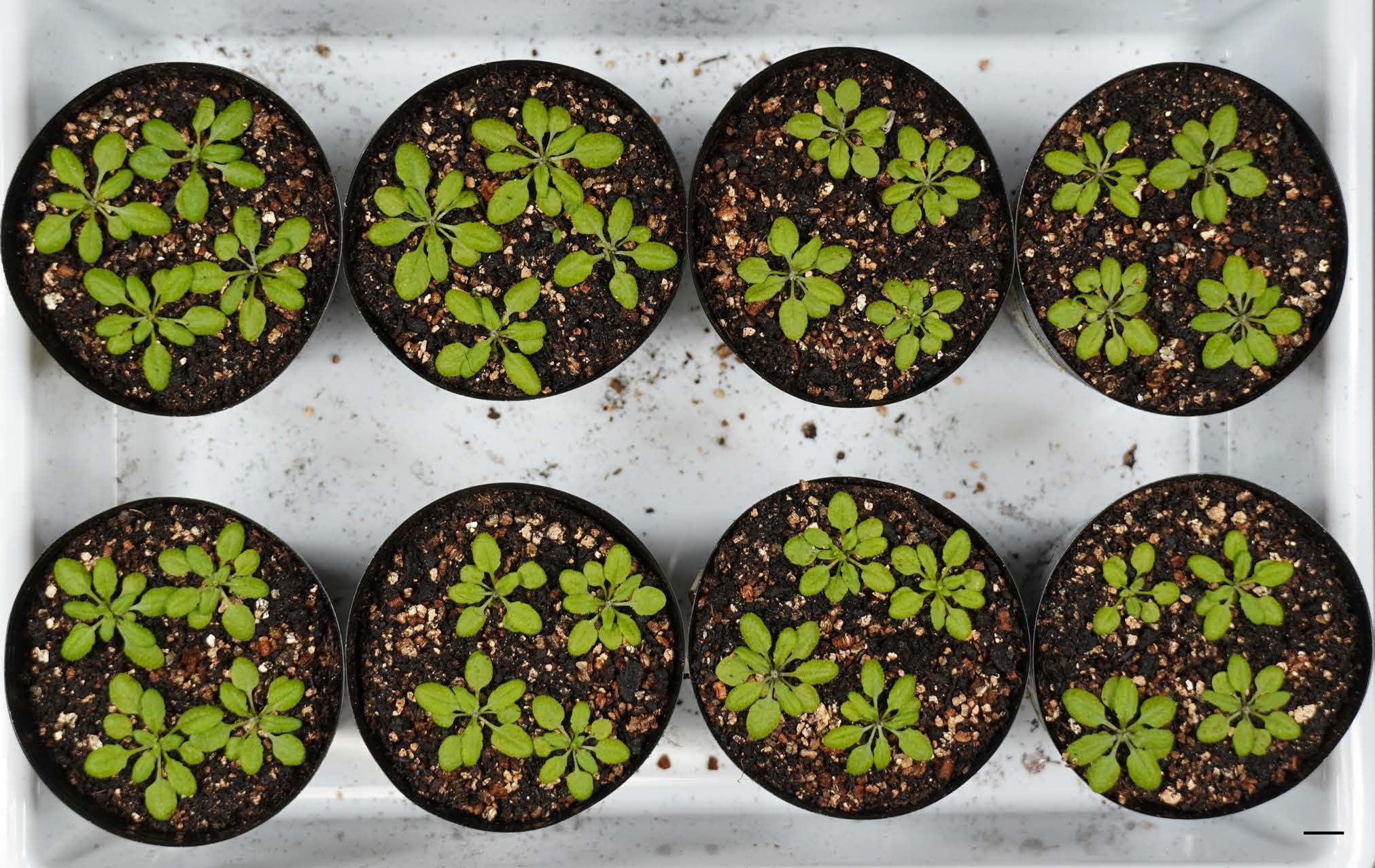

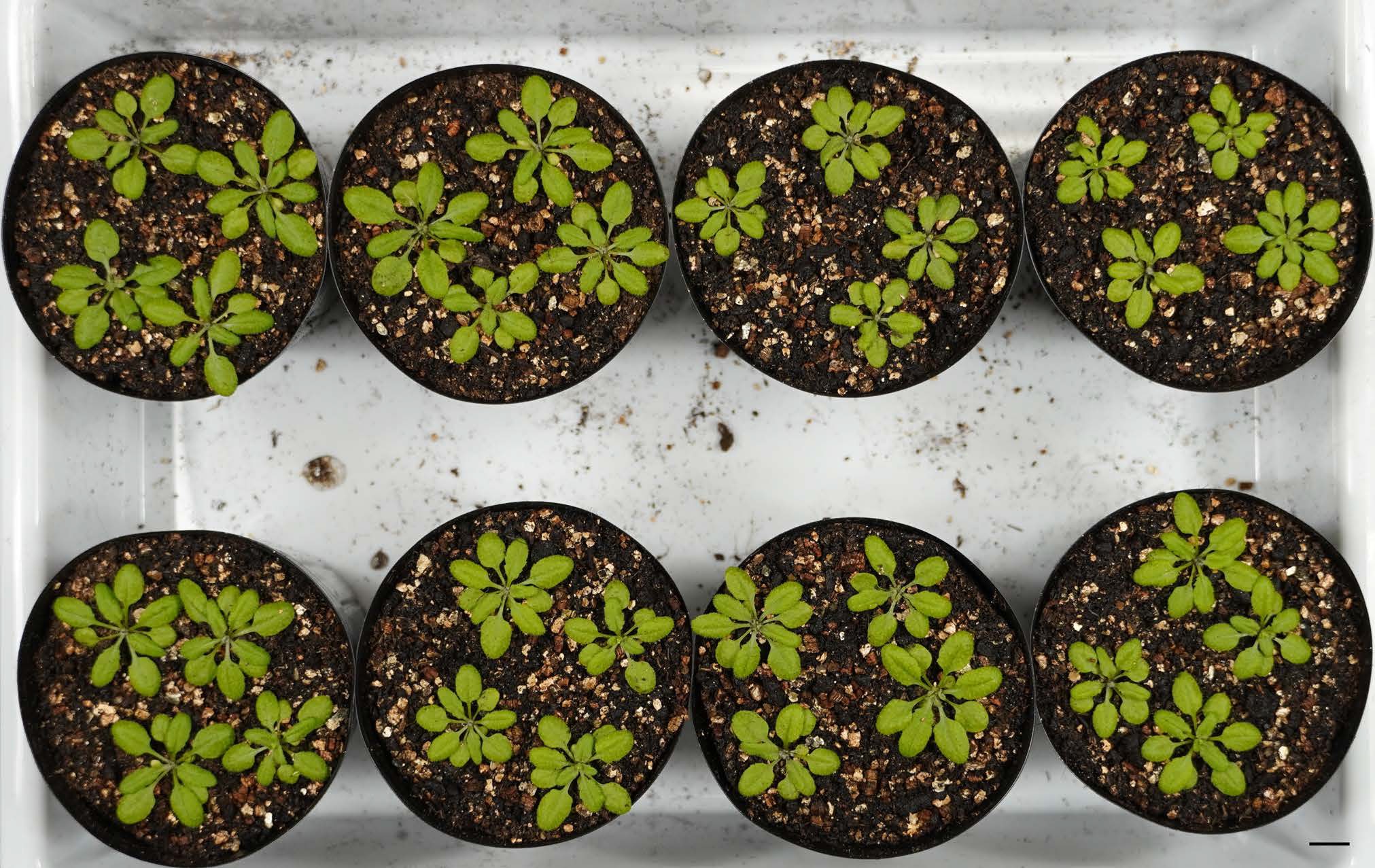


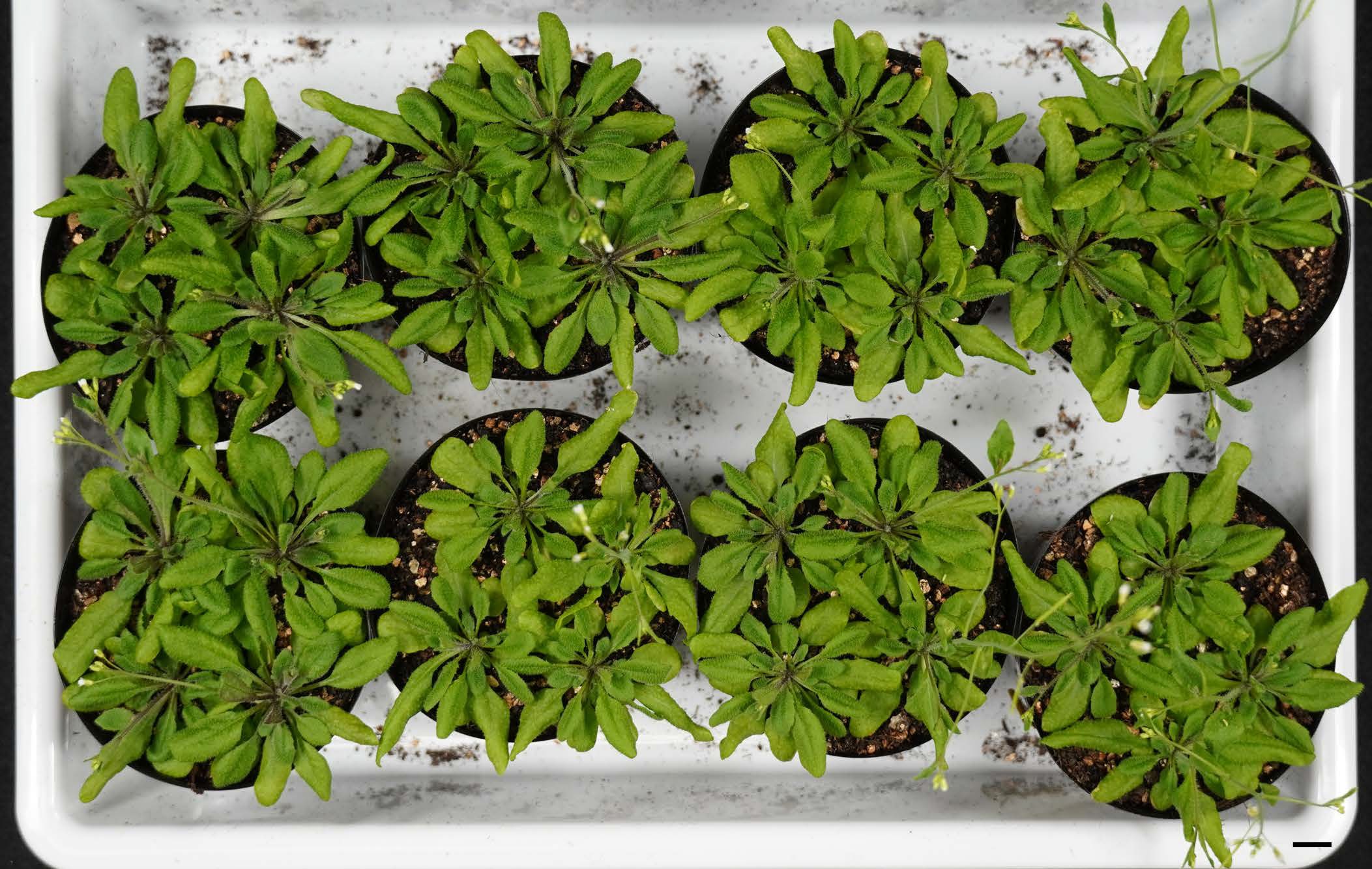

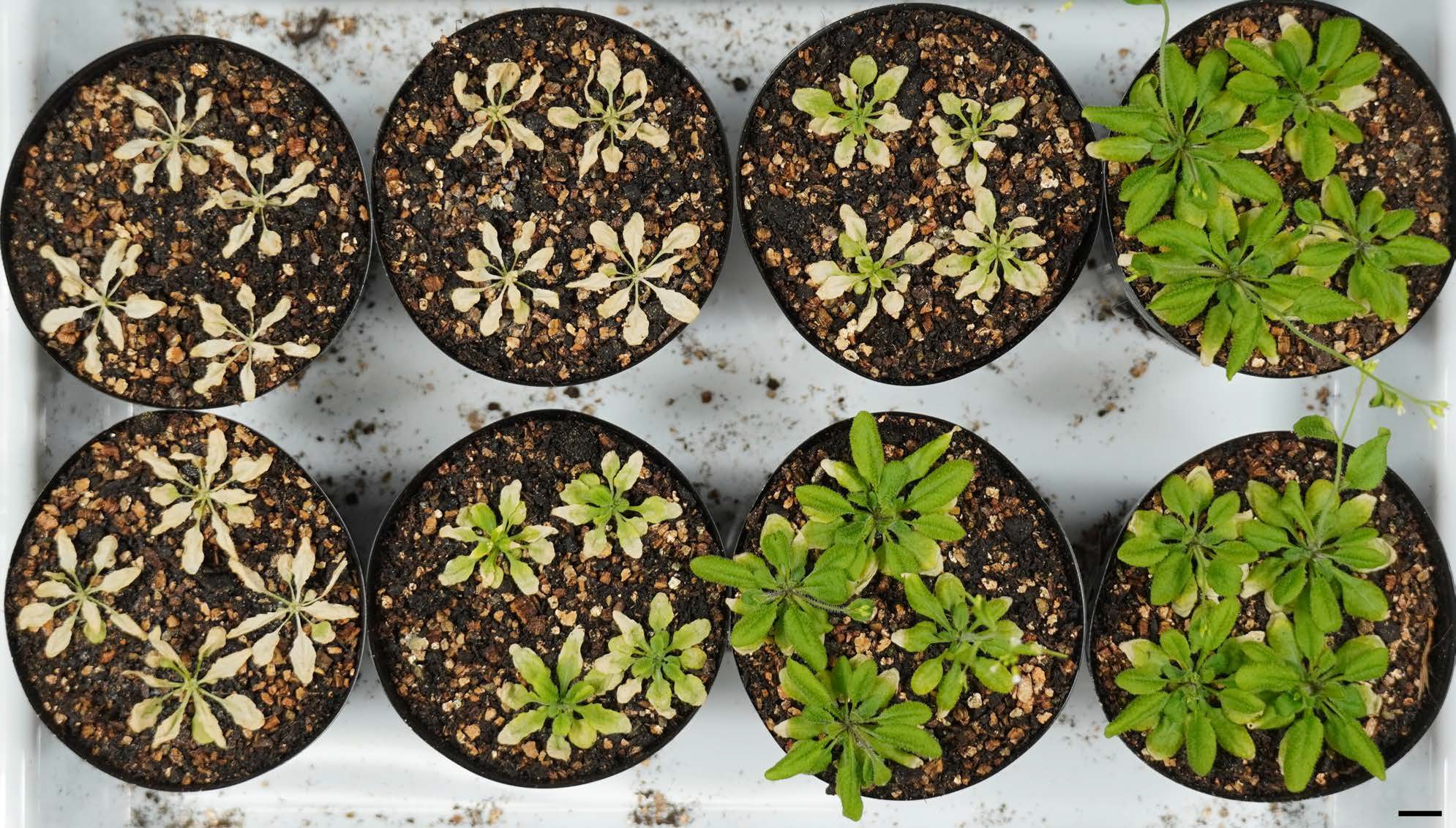

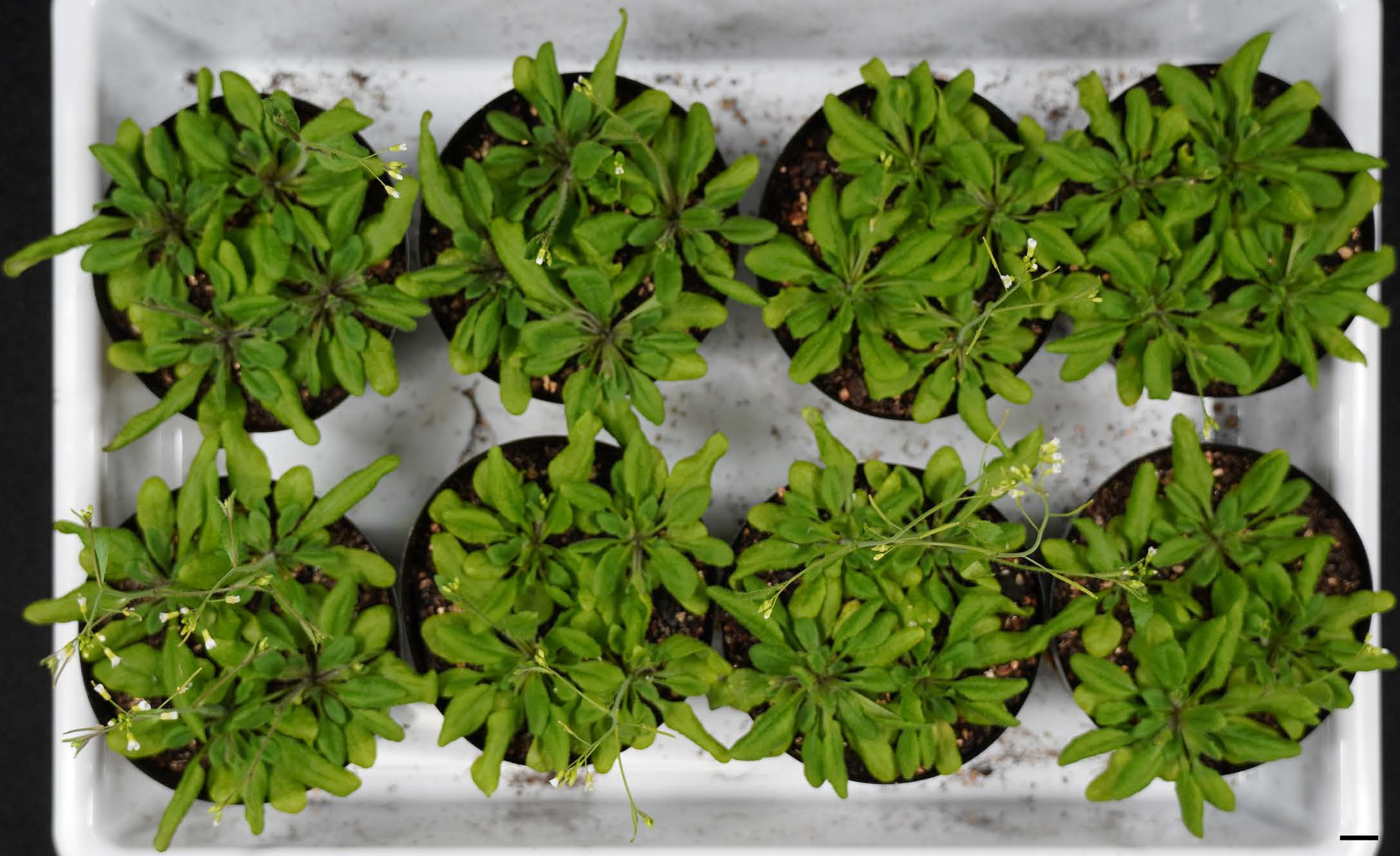

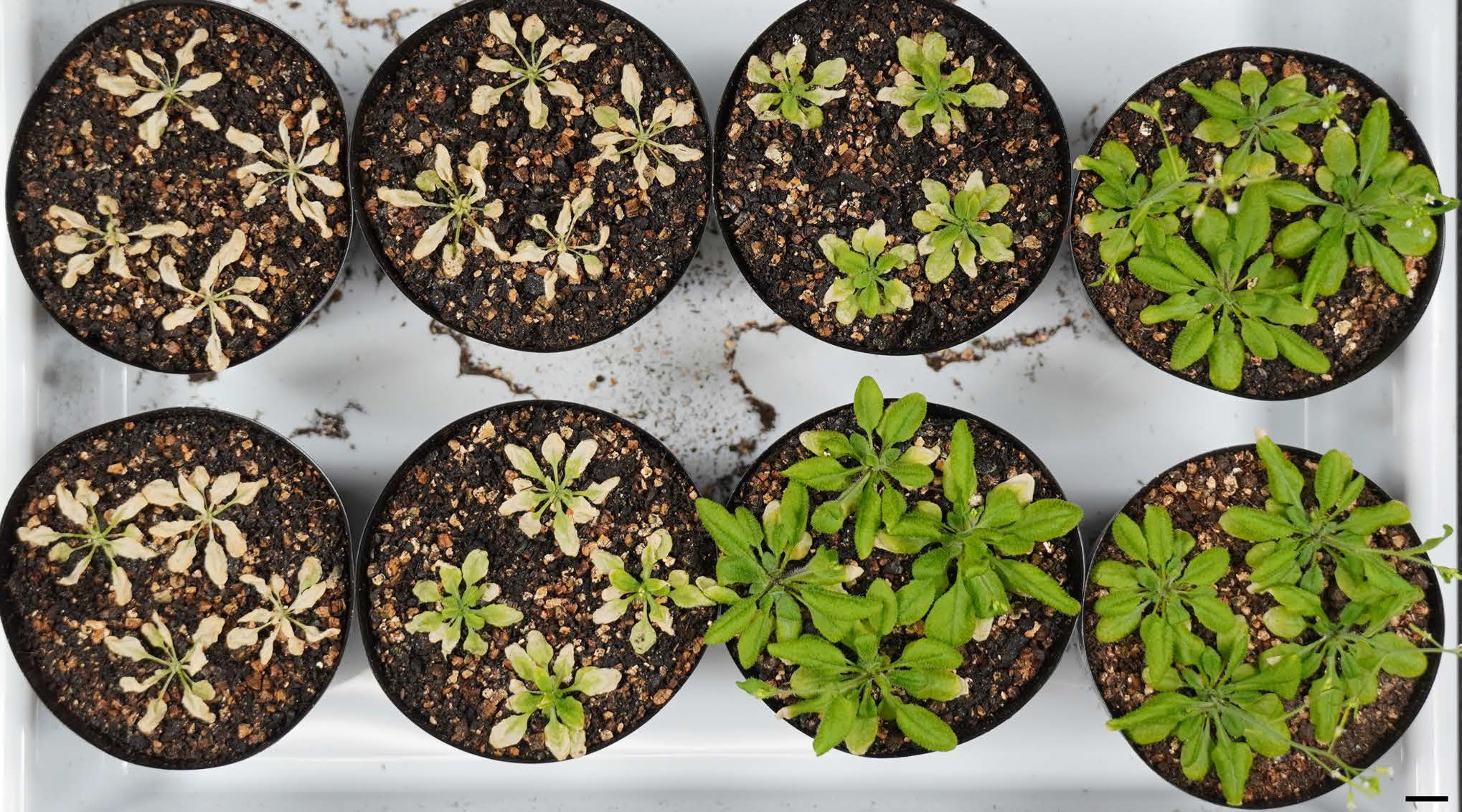
**g** Map of the trays shown in (a) and (b)

**8 days after metribuzin treatment**

**8 days after metribuzin treatment**

| **Col-0** | **A251V**  **Line１** | **A251V**  **Line 4** | **V219I & A251V**  **Line 5** |
| --- | --- | --- | --- |
| **V219I**  **Line 1** | **A251V**  **Line２** | **V219I & A251V**  **Line 3** |  |
| **V219I**  **Line 2** | **A251V**  **Line３** | **V219I & A251V**  **Line 4** |  |

Mutants are in the T3 generation.

Map of the trays shown in (c)-(f)

| **Col-0** | **V219I**  **Line 2** | **A251V**  **Line 4** | **V219I & A251V**  **Line 4** |
| --- | --- | --- | --- |
| **V219I**  **Line 1** | **A251V**  **Line３** | **V219I & A251V**  **Line 3** | **V219I & A251V**  **Line 5** |

Mutants are in the T3 generation.

**Figure S5 Metribuzin treatment on *psbA* mutants in soil.** (a)-(f) Metribuzin (0.5 g L-1) was sprayed on the plants at 25 days after stratification (DAS). Photos at 25 DAS (before metribuzin treatment) and six or eight days after metribuzin treatment are shown. Bars = 1 cm. The data of the replicated experiment is shown in Figure 2c. (g) Maps of the trays.

**a** Experimental replicate 2 **b**


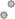


Experimental replicate 2

a

a a

a

a a

a

20

a

a a

a

a a

a

Shoot Dry Weight (mg), without metribuzin

30

15

Leaf Area (cm²), without metribuzin

20

10

10 5

0 0

Col-0

Line 2

Line 3

Line 4

Line 3

Line 4

Line 5

Col-0

Line 2

Line 3

Line 4

Line 3

Line 4

Line 5

A251V V219I & A251V A251V V219I & A251V

1. Experimental replicate 3
2. Experimental replicate 4
3. Experimental replicate 5

10.0

d d

bcd

bcd bc

bcd bd

ac ab

ab a

Leaf Area (cm2), without metribuzin

7.5

10.0

7.5


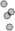

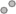


d

de cd

cd d

bde

ae ace ae a ab

Leaf Area (cm2), without metribuzin

10.0

b

Leaf Area (cm2), without metribuzin

7.5 ab

bc ab

ab

ab ab

5.0

5.0

5.0

1. ac a a

2.5

2.5

2.5

0.0

0.0

0.0

Col-0

V219I

Genotype

A250T A251V

V219I & A251V

A250T & A251V

**Figure S6** S**hoot dry weights and leaf areas of *psbA* mutants in soil.** (a) Shoot dry weights four weeks after sowing. The data of Experimental replicate 1 is shown in Figure 2d. (b-e) Leaf areas four weeks after sowing (b) or at 25 days after stratification (c-e). T2 plants (V219I & A251V Lines 1 and 2) were confirmed to have the V219I & A251V double mutations and not to have *ptpTALECD_v2* in the nuclear genome by PCR analyses. The data of leaf areas of Experimental replicate 1 is shown in Figure 2e. Different letters indicate significantly different values (P < 0.05, Tukey-Kramer multiple comparison test). Error bars represent standard deviation. *n* (the number of plants) = 4.

1. Control (without metribuzin)

Y(II) at 100 µmol m-2 s-1

Y(NPQ) at 100 µmol m-2 s-1

Y(NO) at 100 µmol m-2 s-1


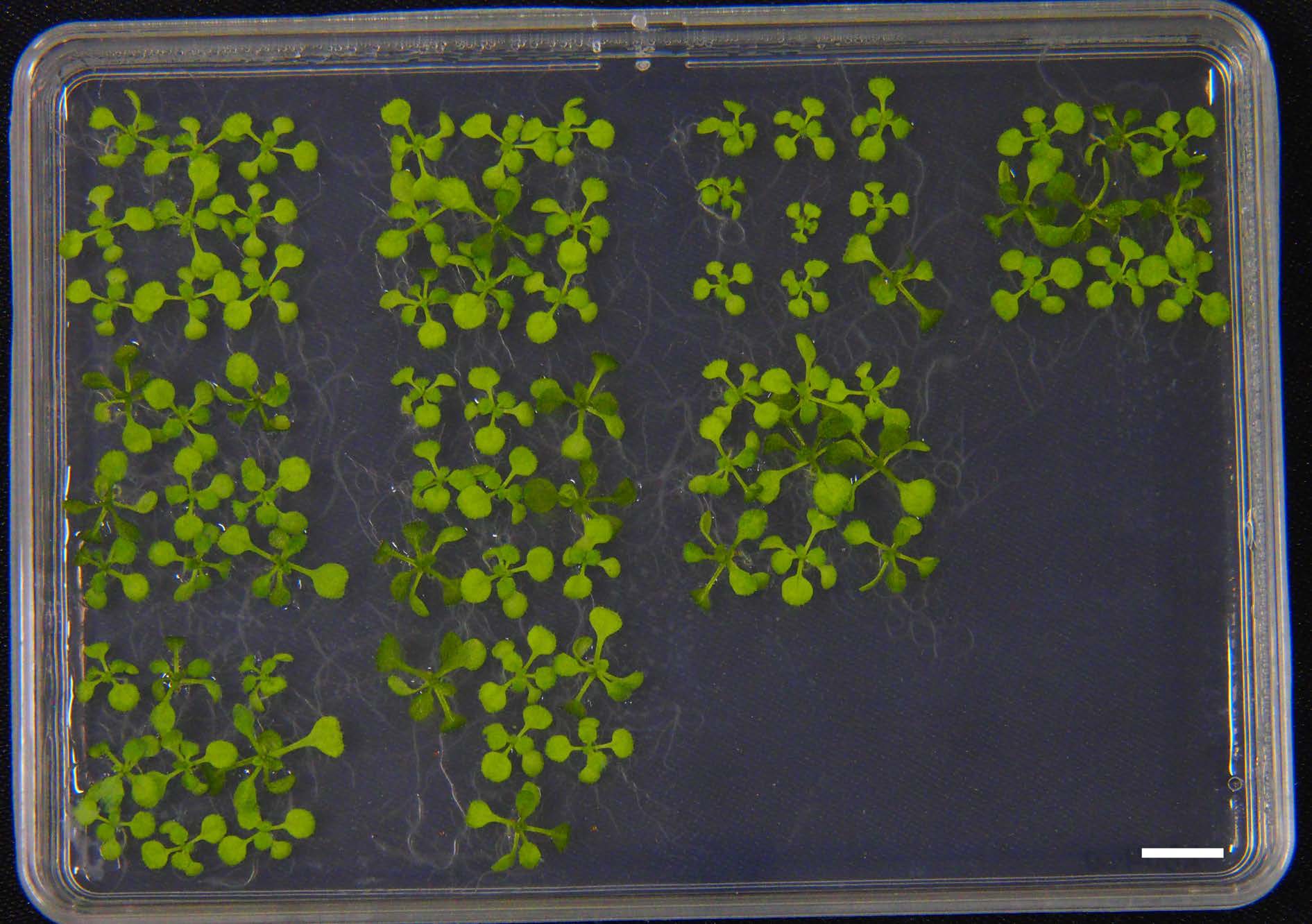

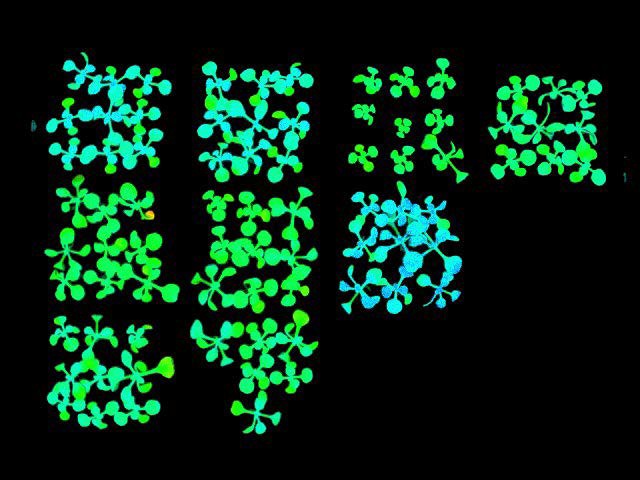

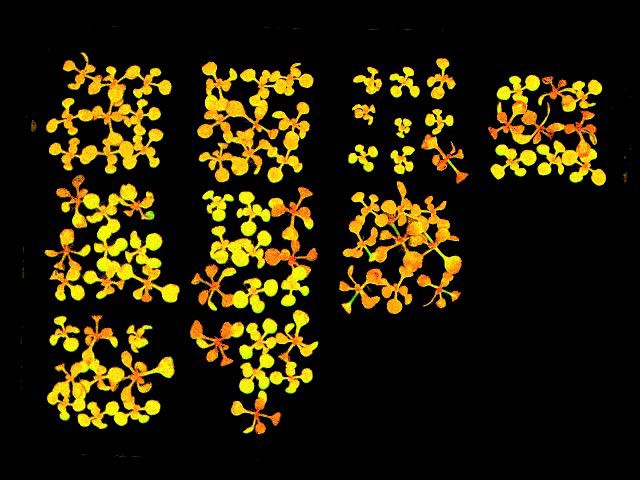

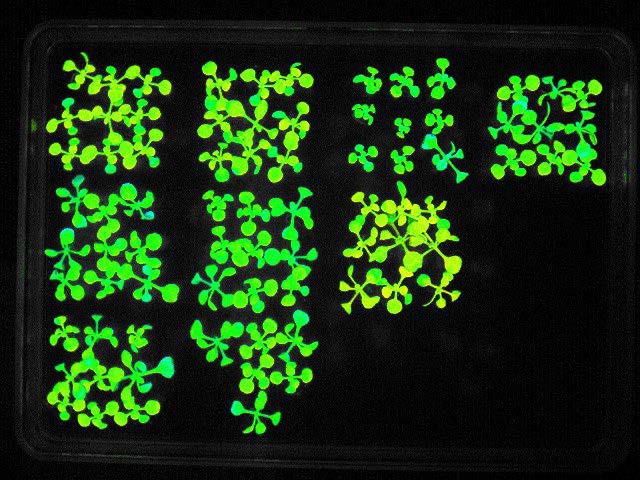


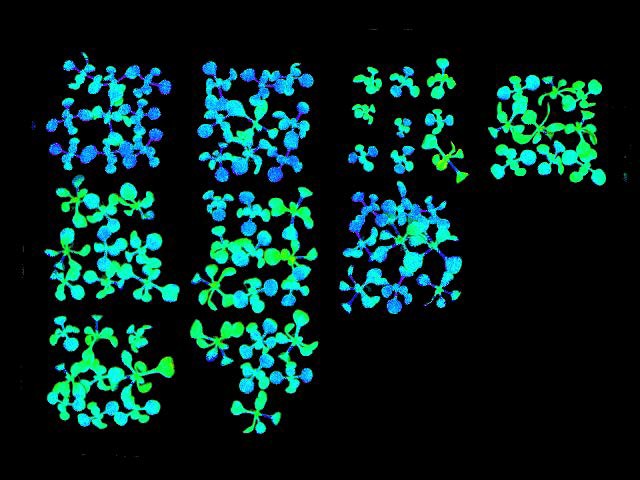

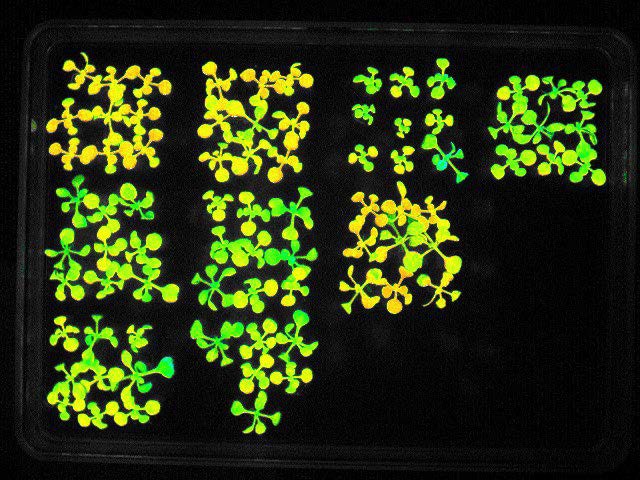

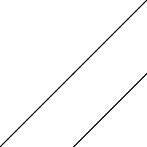
Y(II) at 500 µmol m-2 s-1 Y(NPQ) at 500 µmol m-2 s-1 Y(NO) at 500 µmol m-2 s-1

| **V219I**  **Line 1** | **V219I**  **Line 2** | **A251V**  **Line1** | **A251V**  **Line2** |
| --- | --- | --- | --- |
| **A251V**  **Line3** | **A251V**  **Line4** | **Col-0** |  |
| **V219I & A251V**  **Line 1*** | **V219I & A251V**  **Line 2*** |  |  |


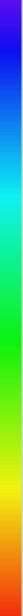
high


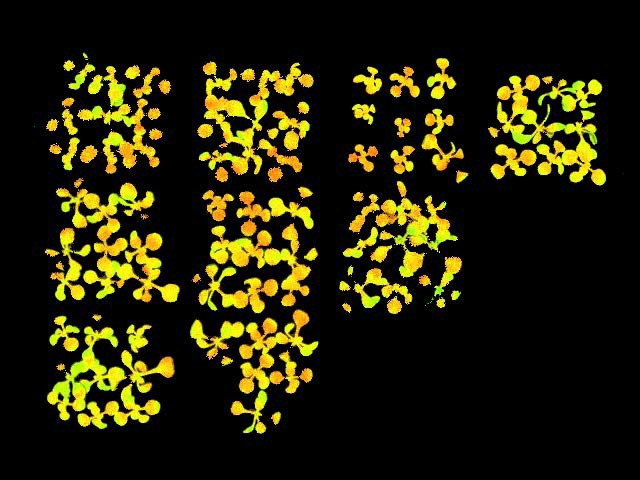
*T2 plants

The other mutants are in the T3 generation.


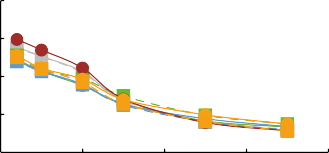
0.8

0.6

Y(II)

0.4

0.2

0.8


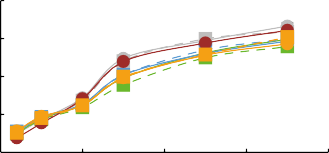
0.6

Y(NPQ)

0.4

0.2

0.8


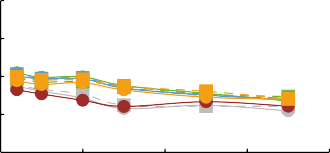
0.6

Y(NO)

0.4

0.2

low

0 0 200 400 600 800 0 0 200 400 600 800 0 0 200 400 600 800

Photon flux density (µmol m-2 s-1)

Photon flux density (µmol m-2 s-1)

Photon flux density (µmol m-2 s-1)


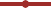

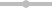
Col-0 V219I Line 1


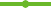

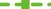
A251V Line 1


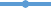

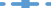
A251V Line 3


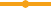

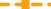
V219I & A251V Line 1


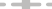
V219I Line 2

A251V Line 2 A251V Line 4

V219I & A251V Line 2

1. 1 mg/L metribuzin


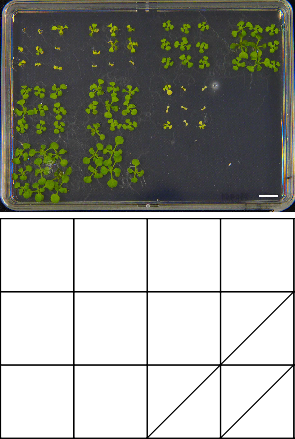


**V219I & A251V**

**Line 2***

**V219I & A251V**

**Line 1***

**Col-0**

**A251V**

**Line4**

**A251V**

**Line3**

**A251V**

**Line2**

**A251V**

**Line1**

**V219I**

**Line 2**

**V219I**

**Line 1**

Y(II) at 100 µmol m-2 s-1 Y(NPQ) at 100 µmol m-2 s-1 Y(NO) at 100 µmol m-2 s-1


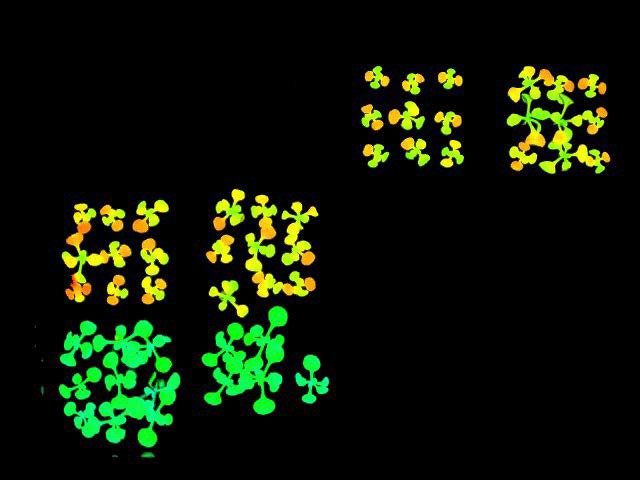

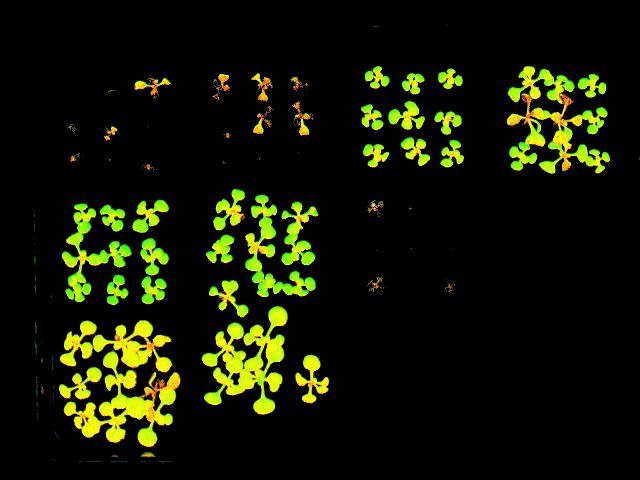

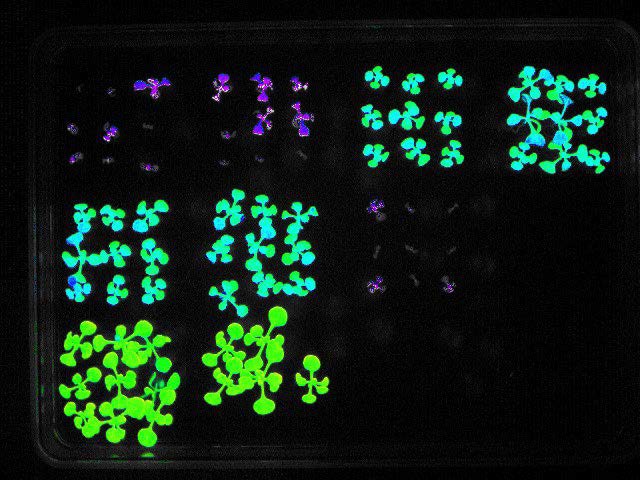


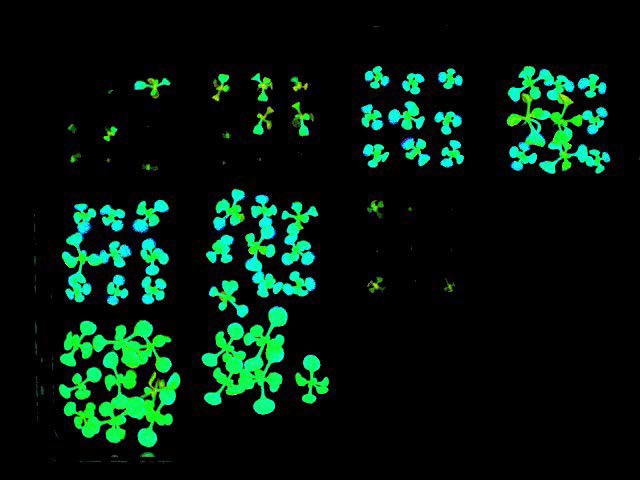

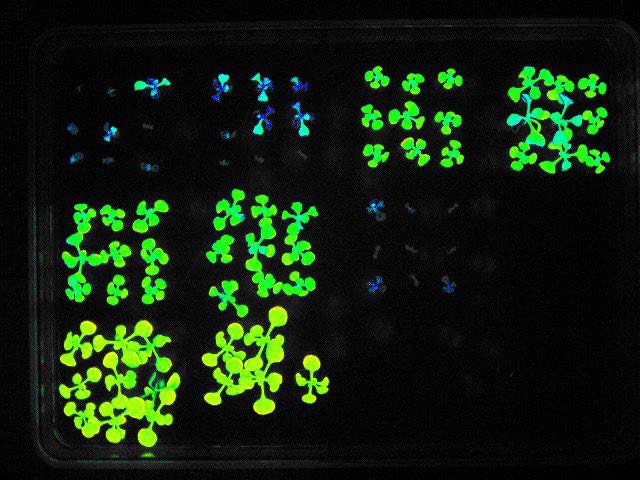
Y(II) at 500 µmol m-2 s-1 Y(NPQ) at 500 µmol m-2 s-1 Y(NO) at 500 µmol m-2 s-1


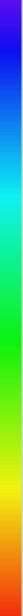
high


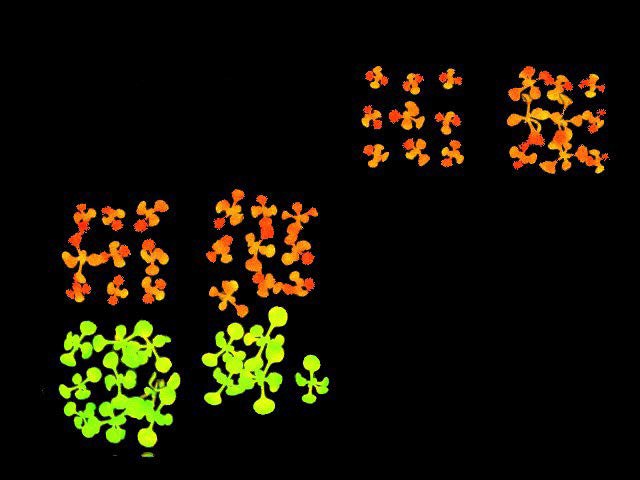
*T2 plants

The other mutants are in the T3 generation.


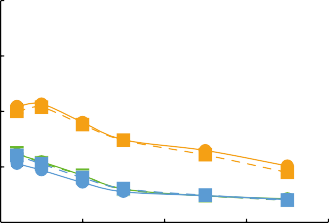
0.8

0.6

Y(II)

0.4

0.2

0.8


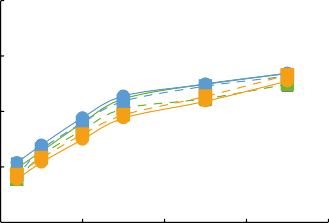
0.6

Y(NPQ)

0.4

0.2

0.8


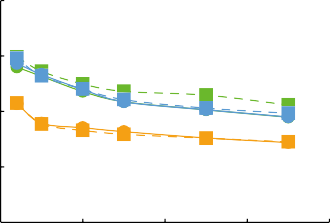
0.6

Y(NO)

0.4

0.2

low

0 0 200 400 600 800

Photon flux density (µmol m-2 s-1)

0 0 200 400 600 800

Photon flux density (µmol m-2 s-1)

0 0 200 400 600 800

Photon flux density (µmol m-2 s-1)


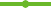

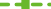
A251V Line 1


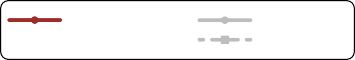


Col-0

V219I Line 1

V219I Line 2


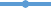

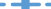
A251V Line 3


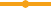

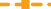
V219I & A251V Line 1

not detected

A251V Line 2 A251V Line 4

V219I & A251V Line 2

**Figure S7 Photosynthetic characteristics of *psbA* mutants in the absence or presence of metribuzin.** The photosynthetic characteristics were analyzed at 12 days after stratification in the absence (a) or presence (b) of metribuzin. After determination of Fv/Fm in the dark, the quantum yield of PSII [Y(II)], that of non-photochemical quenching [Y(NPQ)], and that of other processes including non-regulated heat dissipation and fluorescence emission [Y(NO)] were measured at various light intensities after plants were irradiated for over 5 min to obtain steady-state photosynthesis. Data are means ± standard errors. *n* (the number of plants) = 5-9. Different letters indicate significantly different values (*P* < 0.05, Tukey-Kramer multiple comparison test). ND, not detected; NS, not significant. All the T2 plants (V219I & A251V Lines 1 and 2) were confirmed to have the V219I & A251V double mutations and most of the T2 plants (except for one plant of V219I & A251V Line 1 on the metribuzin- containing medium) were confirmed not to have *ptpTALECD_v2* in the nuclear genome by PCR

analyses.

**a**

a a a a a a a

**0.8**

**0.6**

**0.4**

**Fv/Fm**

**0.2**

**0**

Col-0

Line 2

Line 3

Line 4

Line 3

Line 4

Line 5

A251V V219I & A251V

1. **0.7**

a

ab ab ab

b b

b b

bc bc

c c

b b

bc bc

c c

a

**Col-0**

**A251V Line 2**

**A251V Line 3**

**A251V Line 4**

**V219I & A251V Line 3 V219I & A251V Line 4 V219I & A251V Line 5**

b ab ab

b b b

a

ab ab ab ab

b b

a

ab b ab ab ab ab

**0.6**

**0.5**

**0.4**

**Y(II)**

**0.3**

**0.2**

**0.1**

**0**

**30 74**

**146**

**368**

**595**

**886**

**PAR (µmol photons m2 s-1)**

1. **100**

**Col-0**

**A251V Line 2**

**A251V Line 3**

**A251V Line 4**

**V219I & A251V Line 3**

a

a

ab ab ab ab ab

b

ab ab ab ab

**V219I & A251V Line 4**

**V219I & A251V Line 5**

b b

a

b b b b ab ab

a

c c bc bc b b

a

c c bc bc b b

a b b b ab ab ab

**80**

**ETR (µmol electrons m-2 s-1)**

**60**

**40**

**20**

**0**

**30 74**

**146**

**368**

**595**

**886**

**PAR (µmol photons m2 s-1)**

**Figure S8 Photosynthetic characteristics of *psbA* mutants cultivated in soil conditions without metribuzin.** (a) Fv/Fm and (b) Y(II) at various light intensities. (c) The electron transfer rate (ETR) of PSII were calculated as ETR = 0.5 × abs I × Y(II), where 0.5 is the fraction of absorbed light between PSII and PSI (assuming they are equal), and abs I is absorbed irradiance taken as 0.84 of incident irradiance. Data are means ± standard errors. *n* (the number of plants) = 7. Different letters indicate significantly different values (P < 0.05, Tukey-Kramer multiple comparison test).
